# Geological evidence reveals a staircase pattern in Earth’s rotational deceleration evolution

**DOI:** 10.1073/pnas.2317051121

**Published:** 2024-08-06

**Authors:** He Huang, Chao Ma, Jacques Laskar, Matthias Sinnesael, Mohammad Farhat, Nam H. Hoang, Yuan Gao, Christian Zeeden, Hanting Zhong, Mingcai Hou, Chengshan Wang

**Affiliations:** ^a^State Key Laboratory of Oil and Gas Reservoir Geology and Exploitation, Institute of Sedimentary Geology, Chengdu University of Technology, Chengdu 610059, China; ^b^Key Laboratory of Deep-time Geography and Environment Reconstruction and Applications of Ministry of Natural Resources, Chengdu University of Technology, Chengdu 610059, China; ^c^Institut de mécanique céleste et de calcul des éphémérides, CNRS, Observatoire de Paris, Paris Sciences and lettres University, Sorbonne Université, Paris 75014, France; ^d^Department of Geology, School of Natural Sciences, Trinity College Dublin, The University of Dublin, Dublin 999015, Ireland; ^e^State Key Laboratory of Biogeology and Environmental Geology, China University of Geosciences (Beijing), Beijing 100083, China; ^f^LIAG-Leibniz Institute for Applied Geophysics, Hannover 30655, Germany

**Keywords:** Earth–Moon system, Earth’s rotation, cyclostratigraphy, tidal dissipation, geological evolution

## Abstract

The Earth’s rotation has been decelerating throughout its history due to tidal dissipation, but the variation of the rate of this deceleration through time has not been established. We present a detailed analysis of eight geological datasets to constrain the Earth’s rotational history from 650 to 240 Mya. The results allow us to test physical tidal models and point to a staircase pattern in the Earth’s deceleration from 650 to 280 Mya. During this time interval, the Earth–Moon distance increased by approximately 20,000 km and the length of day increased by approximately 2.2 h. Specifically, there are two intervals with high Earth rotation deceleration, 650 to 500 Mya and 350 to 280 Mya, separated by an interval of stalled deceleration from 500 to 350 Mya. The interval with stalled deceleration is attributed mainly to reduced tidal dissipation due to the continent-ocean configuration at the time, not to changes in Earth’s dynamical ellipticity from continental assembly or glaciation. Modeling indicates that, except for the very recent time, tidal dissipation is the main driver for decelerating Earth rotation. One potential implication of our findings is that the Earth’s tidal dissipation, along with Earth’s rotation deceleration, may play a role in the evolving Earth.

## Significance

Astronomically forced climate change is recorded in the Earth’s cyclic sedimentary record (“cyclostratigraphy”). These records can inform on past dynamics of the Solar system and Earth rotation. Here, we reconstruct the evolution of Earth’s rotational deceleration during the Paleozoic Era based on new analysis of selected high-quality cyclostratigraphy datasets. A staircase pattern in deceleration from 650 to 280 Mya is identified and attributed to the occurrence of tidal dissipation resonance. Modeling indicates that the development of major glaciations during the Paleozoic had a negligible impact on Earth’s rotation.

Earth transfers angular momentum through tidal dissipation to the orbit of the Moon, resulting in an increasing of Moon’s orbital radius and a deceleration of Earth’s rotation (1). The deceleration has changed over time and its rate has not been constant (2–5). The Earth’s rotation influences its axial precession, which has a present average rate of 50.475838 arcseconds per year (arcsec/y, denoted as *k*) (6). Thus, the axial precession can be used for reconstruction of the Earth’s rotation history. At present, the evolution of the Earth’s rotation through time is otherwise largely unknown. Lunar laser ranging (LLR) observations of today’s lunar recession rate (ca. 3.83 cm/y) (7) and the age of the Moon [ca. 4.425 billion years ago (Ga)] (8) provide two constraints on lunar recession history. However, backward projection of the present lunar recession rate predicts collision between the Moon and Earth at ~1.5 Ga (9, 10), which is incompatible with the Moon’s age inferred from radioisotopic dating (8, 11, 12). Numerous studies have proposed various solutions to solve this paradox with analytical models, numerical simulations, and geological data (10, 13–16). However, theoretical tidal models are limited in describing dissipative processes, and geological data are necessary for constraining theoretical models.

**Fig. 1.**
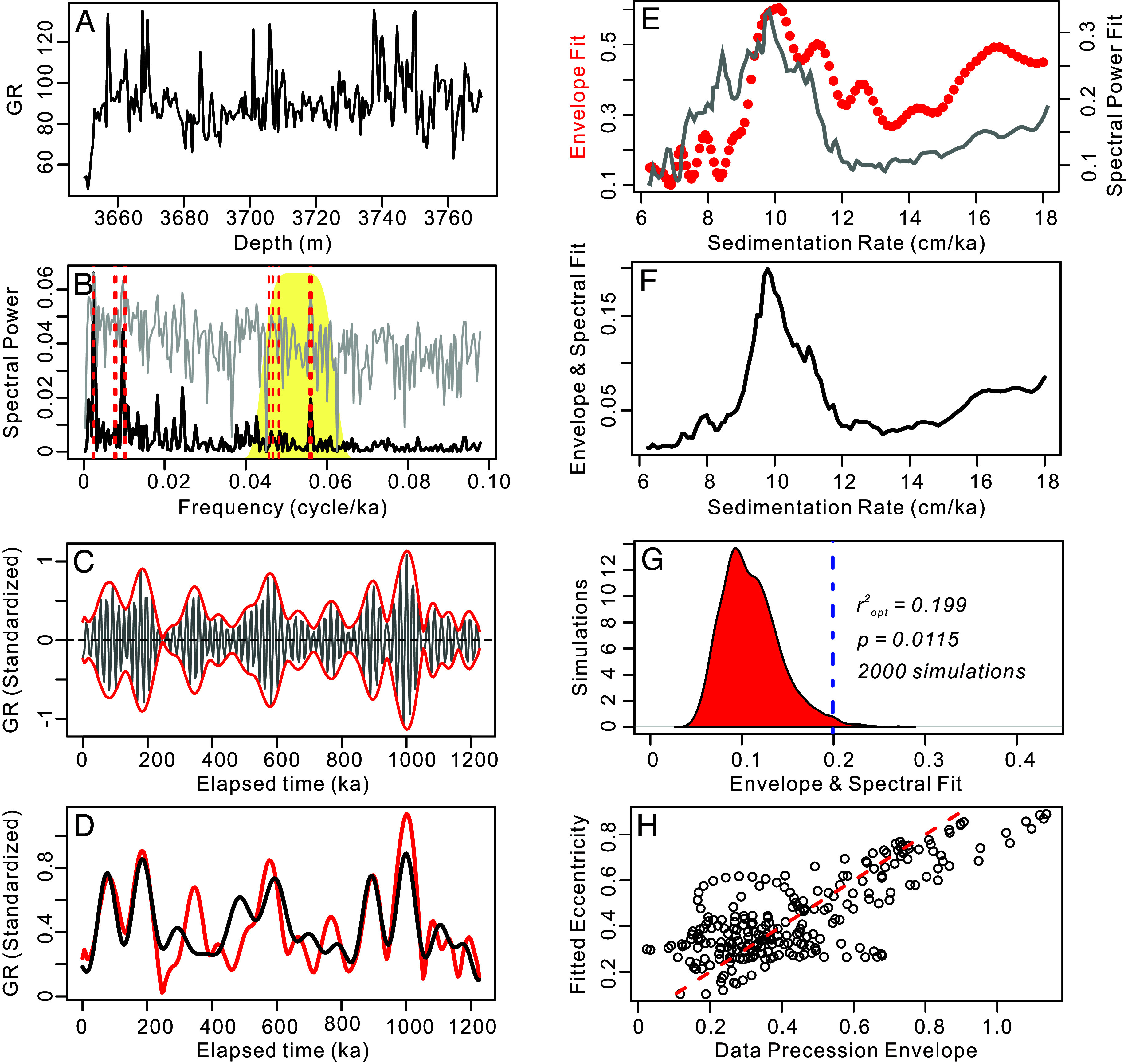
TimeOpt and TimeOptSim analyses of the cyclostratigraphic dataset from the Permian Lucaogou Formation (ca. 290 Ma) at Junggar Basin, China (33). An identical analytical procedure was applied to the remaining seven geological datasets (*SI Appendix*, Figs. S1–S7). (*A*) The gamma ray (GR) data series. (*B*) Periodogram for the GR data (black line = linear spectrum; gray line = log spectrum). The yellow shaded region indicates the portion of the spectrum bandpass filtered for evaluation of the climatic precession amplitude envelope. The vertical dashed red line indicates the eccentricity and climatic precession target frequencies. (*C*) Extracting the band-passed climatic precession signal (black), and the data amplitude envelope (red) determined via Hilbert transform. (*D*) Comparison of the data amplitude envelope (red) and the TimeOpt reconstructed orbital eccentricity model (black). (*E*) Squared Pearson correlation coefficient for the amplitude envelope fit and the spectral power fit as a function of sedimentation rate. (*F*) Combined envelope and spectral power fit at each evaluated sedimentation rate. (*G*) Summary of 2000 Monte Carlo simulations with AR1 surrogates. (*H*) Cross-plot of the data amplitude envelope and the TimeOpt-reconstructed orbital eccentricity model in panel “*D*”; the dashed red line is the 1:1 line.

Over the past 60 y, a series of geological archives has been analyzed to estimate, for example, the number of Earth days per lunar month from tidalites (5, 17, 18), and the number of days per solar year from daily growth rings of fossils (2, 19–22). Although the analysis of tidalites and fossils has improved our understanding of Earth’s deceleration (23), tidalites, and fossils are scarce and limited through geological history and exhibit large uncertainties in their interpretation (5, 23–29). By contrast, recent developments in cyclostratigraphy have enabled the extraction of Earth’s astronomical parameters from astronomically forced stratigraphy with robust quantitative methods (30–32). Consequently, a growing number of values for past *k* are being reported (e.g., refs. 30–36). Recently, cyclostratigraphy has emerged as the most robust geological archive for deciphering past changes in Earth’s rotation and lunar recession (24), especially when a match between the recorded amplitudes of the climatic precession and orbital eccentricity can be demonstrated (Fig. 1). Continued gathering of reliable geological data to test theoretical tidal models is particularly crucial for times that may show past oceanic tidal resonances (16). Here, we use the Markov-Chain Monte Carlo (MCMC) Bayesian inversion method developed by ref. 30, (TimeOptMCMC; see *Materials and Methods*) to estimate *k* values from eight cyclostratigraphic time series covering ages ranging from 570 Ma to 245 Ma (33, 37–43) (Fig. 1 and *SI Appendix*, Figs. S1–S8 and Table S1). These *k* values, along with other published *k* values (*SI Appendix*, Fig. S9 and Table S1), constrain Earth’s rotation history and serve as an independent data basis to test theoretical tidal models.

## Results

### New Precession Frequency (*k*) Estimates.

**Fig. 2.**
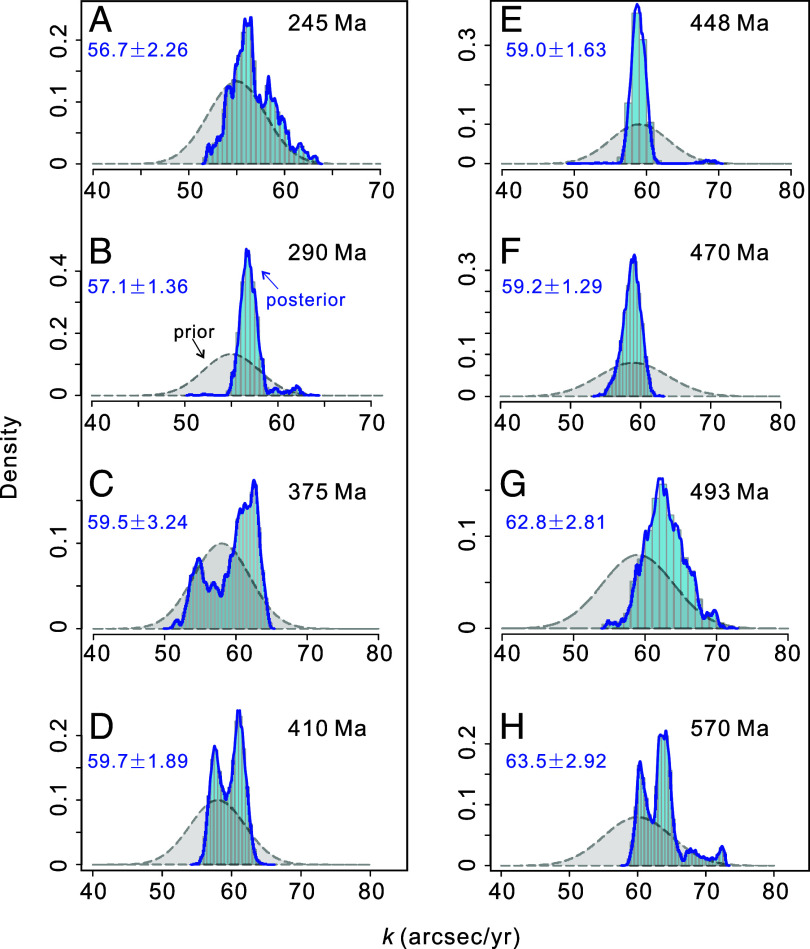
Prior and posterior distributions of the *k* values (*A–H*) from TimeOptMCMC analysis of eight sets of cyclostratigraphic data (33, 37–43). Shaded gray areas indicate the prior distributions, and blue-shaded histograms indicate the posterior distributions obtained by Markov-Chain Monte Carlo sampling.

Starting from a broad literature review, we compiled a suite of published cyclostratigraphic data series (*SI Appendix*, Table S1) that were suitable for TimeOptMCMC analysis (*SI Appendix*, Table S1 and *Materials and Methods*). We selected eight cases that are presented in more detail in the *Materials and Methods* and range from the Ediacaran (570 Ma) to the early Middle Triassic (245 Ma) (33, 37–43). The TimeOptMCMC analyses on these cyclostratigraphic data used prior distributions of the sedimentation rate (SR) following the original publications and were further independently constrained by our TimeOpt and TimeOptSim analyses (*SI Appendix*, Fig. S8 and Table S3), while the prior *k* ranges were obtained from the tidal model of Waltham (W15 model; *Materials and Methods* and *SI Appendix*, Table S2) (13). The TimeOptMCMC results of the eight cyclostratigraphic time series are shown in Fig. 2 and *SI Appendix*, Table S3. The blue histograms depict the posterior distributions of the *k*, while prior distributions are in gray (Fig. 2). The posterior distributions are more confined compared to the prior distributions. This outcome signifies successful optimization of *k* (Fig. 2), SR (*SI Appendix*, Fig. S8), and the fundamental secular frequencies *g_i_* (i = 1, 2, 3, 4, 5; 1 = Mercury, 2 = Venus, 3 = Earth, 4 = Mars, 5 = Jupiter) by TimeOptMCMC. The mean value and SD (σ) of *k* was calculated from the post-burn-in results of the MCMC simulations (Fig. 2 and *SI Appendix*, Table S3). Using the estimated *k* values, we derive the corresponding Earth–Moon distance (EMD), the length of the day (LOD) and Earth’s obliquity angle according to the model of Farhat et al. (16) using the tool provided on the *AstroGeo* website (http://www.astrogeo.eu/) (*SI Appendix*, Table S3). For example, TimeOptMCMC analysis generated a posterior distribution that determines *k =* 56.7 ± 2.26 arcsec/y at 245 Ma (Fig. 2*A*). This *k* value corresponds with an EMD of 374.0 (+3.36/−3.22) thousand kilometers, LOD of 22.6 (+0.46/−0.45) h and an average obliquity angle at 22.6 (+0.21/−0.21) degrees (*SI Appendix*, Table S3).

### Trend Changes in *k* through Time.

**Fig. 3.**
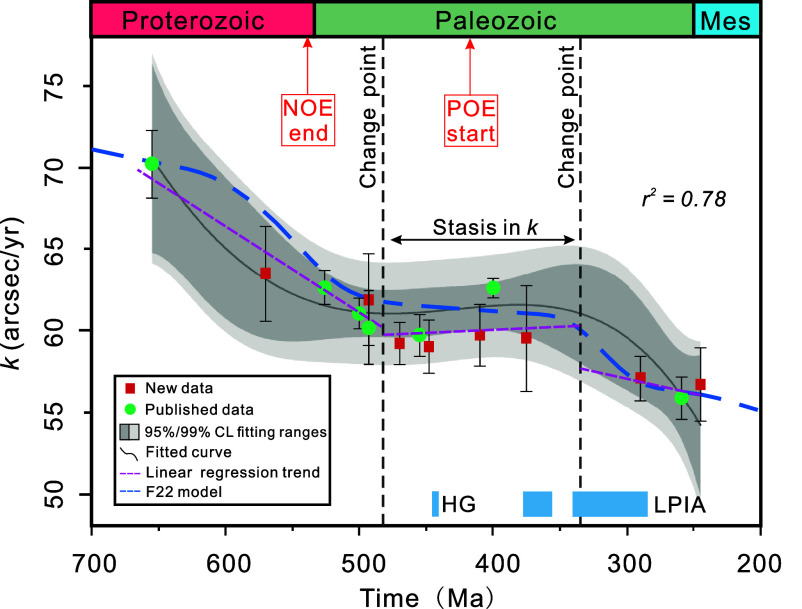
Cyclostratigraphic reconstruction of *k* and its trends through time. The reported uncertainties are one SD (1σ). The gray shaded and light gray shaded areas indicate the 95% and 99% confidence levels for the fitted data range, respectively. The dark-gray curve represents the quartic polynomial fitting results for these data (*r*^2^ = 0.78). The black dotted lines represent the outputs of the change-point analysis, which divide the data into three groups. The purple dotted curves represent the linear regression trends for the data points within each of the three intervals. The blue dashed line represents the evolution of average *k* in the F22 tidal model (16). The red arrows indicate the end of NOE at ca. 540 Ma (46) and the start of the POE at ca. 420 Ma (47–49). Mes: Mesozoic; LPIA: Late Paleozoic Ice Age; HG: Hirnantian Glaciation; NOE: Neoproterozoic Oxygenation Event; POE: Paleozoic Oxygenation Event.

We integrated our new dataset with other published cyclostratigraphically derived *k* values spanning from ca. 700 Ma to 200 Ma (Fig. 3). In a similarly way to ref. 44, we employed change-point analysis (45) (*Materials and Methods*) to identify trends in evolution among the reconstructed *k* values (Fig. 3). There are three distinct groups which reveal two notable changes in *k* (Fig. 3). The first substantial change occurred between ca. 650 Ma and ca. 480 Ma, characterized by a relative rapid decrease in *k.* Between 480 Ma and 350 Ma, the trend was remarkably flat and followed by a second relative fast decrease in *k* between 350 Ma and 280 Ma (Fig. 3). The first fast decrease comprises six data points, indicating a linear deceleration in *k* of approximately 0.059 arcsec/y/My, while the second decrease consists of three data points and exhibits a linear deceleration rate in *k* of ca. 0.0068 arcsec/y/My, which is a lower rate compare to the 650 Ma to 480 Ma decrease. The newly acquired results together with the published data thus indicate a staircase pattern in Earth’s deceleration from 700 Ma to 200 Ma (Fig. 3 and *SI Appendix*, Fig. S10).

## Discussion

### Comparison of Geological Constraints with Tidal Models.

**Fig. 4.**
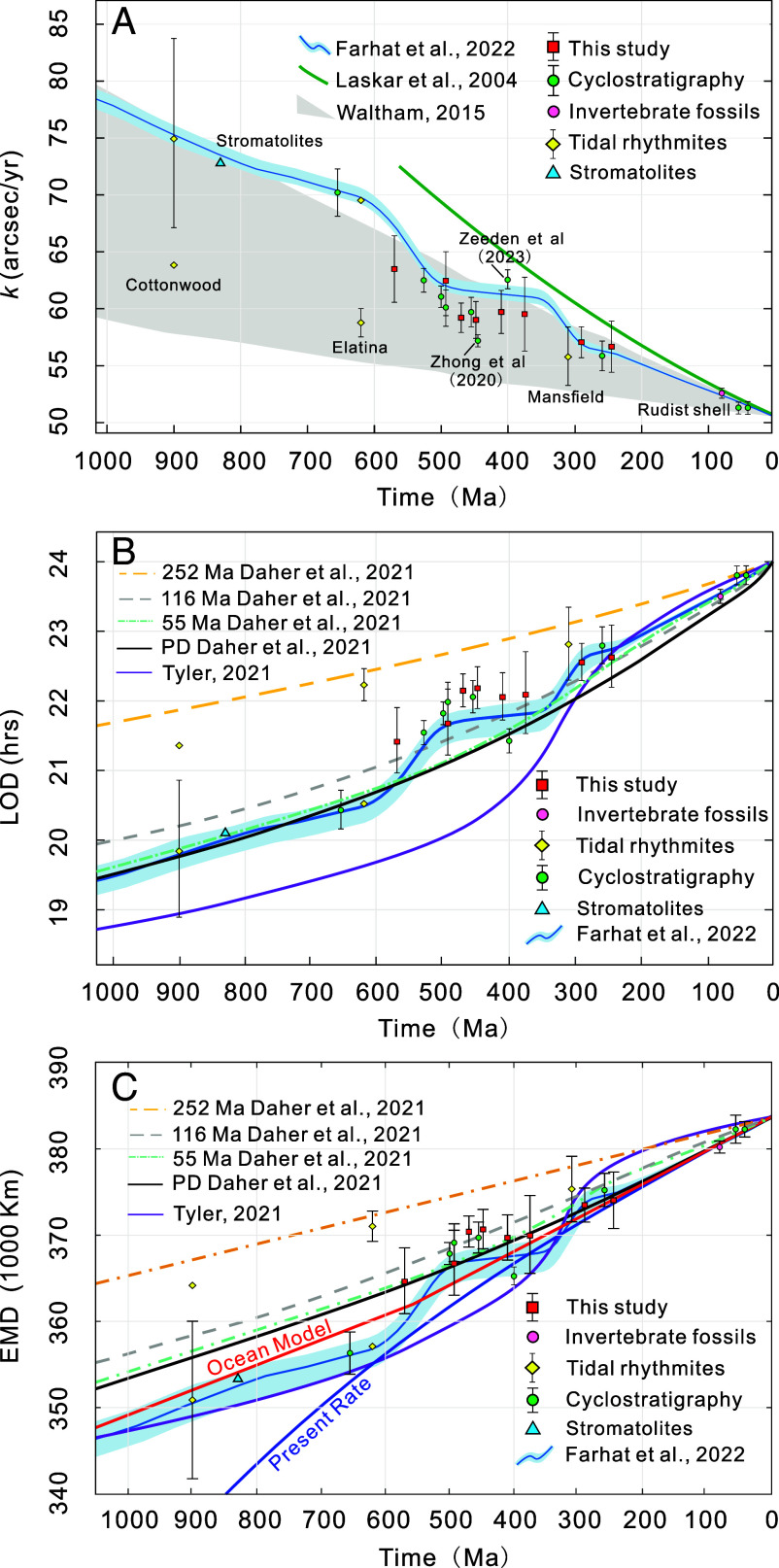
Comparison of *k*, LOD, and EMD with tidal model predictions over the past 1 Ga. (*A*) The estimated Earth’s axis precession frequency versus the astronomical models, where the green line shows Laskar 2004 model (equation 40 in ref. 6), the blue curve with narrow uncertainty is from ref. 16, the gray area delineates the uncertainty given by Waltham's 2015 model (13). (*B*) Comparison of the reconstructed LOD with tidal models (14–16). Note: the D21 model (14) has four tidal evolution solutions based on present-day (PD) ocean basin geometry and 55 Ma, 116 Ma, and 252 Ma reconstructed basin paleogeometries. (*C*) Comparison of the reconstructed EMD with tidal models (14–16), the red line represents the ocean model from ref. 10, and the blue line indicates the present rate of tidal dissipation. *k*: Earth’s axial precession frequency; LOD: the length of the day; EMD: Earth–Moon distance.

Previous studies have proposed a series of models to reconstruct the evolution of the Earth–Moon system based on tidal theory (6, 10, 13–16, 50, 51). These models vary in their underlying assumptions, constraints, and approaches for obtaining a tidal solution. Consequently, they offer a wide range of possible evolutionary tracks of the Earth–Moon system (Fig. 4*A*). Geological observations provide an independent way to constrain the Earth–Moon evolution and test these models. In what follows, we compare these geological findings with five models: the Laskar model (La04) (6), Waltham model (W15) (13), Tyler model (T21) (15), Daher model (D21) (14) and Farhat model (F22) (16) (Fig. 4 *B* and *C*).

The La04 tidal model is based on a constant time lag assumption (52), where the time it takes the Earth to establish its equilibrium state after the lunar tidal stress is fixed. This assumption is valid when describing the system at present and in the recent past, but fixing the time lag over long-term geological timescales is unrealistic given the evolving response of the paleo-oceans. The present state of the ocean system involves an anomalously high tidal dissipation, and so the La04 model overestimates the lunar recession rate in the past. Thus, *k* in the La04 model shows a higher value in comparison with the rest of the models, as well as the geological records (Fig. 4*A*). Waltham (13) reconstructed the history of Earth–Moon separation by employing two fixed endpoints, 384,000 km for the PD and 30,000 km (Roche limit distance) at 4.5 Ga. Thus, the W15 model reports a higher degree of uncertainty in determining *k* due to the limited availability of effective constraints. Consequently, nearly all of the geological records fall within *k* ranges formulated by the W15 model, but we note that these data are more concentrated toward the higher end of the range. Those that do not fall within the model’s range are always above it (Fig. 4*A*). Therefore, the W15 model overestimates the lunar recession rate in the considered time interval, leading to a significant lower limit error in its estimation of the Earth–Moon system’s evolution.

Recent advances in tidal theory, especially for fluid tides, have facilitated the formulation of more refined and physically grounded models. Current state-of-the art models are T21 model (15), D21 model (14), and F22 model (16) (Fig. 4*B*). The T21 model employs a global ocean configuration that persists throughout the lifespan of the Earth–Moon system. It is parameterized by two free variables: an effective oceanic thickness and a timescale of tidal dissipation (15). These two parameters were constrained by fitting the reconstructed system history to the geological data available at the time (which mainly correspond to tidal rhythmites and paleontological clocks). Through comparison with these geological data, we find that the T21 model exhibits a good fit over the past 300 Ma, while beyond 300 Ma, the model results show an increasing discrepancy with geological data (Fig. 4 *B* and *C*). By contrast, Daher et al. (14) used a numerical approach to compute the tidal solution by using four different ocean geometries: a present-day (PD) ocean basin geometry and 55 Ma, 116 Ma, and 252 Ma reconstructed basin paleogeometries. The PD continental configuration and mean sea level value result in unusually larger tides both in open-ocean and coastal regions than most periods of geological history (14, 53). The D21–PD tidal dissipation rate overestimates the past tidal dissipation throughout the Phanerozoic, while during 1000 Ma to 600 Ma, it is similar to PD conditions (Fig. 4 *B* and *C*). D21–55 and D21–116 exhibit a similar trend to D21-PD but demonstrate a better fit with the geological data for the past 100 Ma (Fig. 4 *B* and *C*). D21–252 underestimates past tidal dissipation rates, resulting in a longer LOD and EMD than indicated by geological observations (Fig. 4 *B* and *C*). Green et al. (53) also modeled the tidal energy at 252 Ma, and found that the total dissipation rate was much lower than present day.

Farhat et al. (16) presented a semianalytical physical tidal model that utilizes two parameters to characterize the ocean: the average ocean depth and a dissipation factor. These parameters were tuned such that the reconstructed tidal history fits well with the current lunar recession rate and the Moon’s age. While geological data were not incorporated into the model’s development, the latter independently aligns well with *k* estimates, particularly in concordance with estimates derived from cyclostratigraphy (16, 24). In this study, we also see a higher degree of similarity between our new *k* estimates and previously published geological data and the F22 model compared to the other theoretical models (Fig. 4 and *SI Appendix*, Fig. S9). In the F22 model, the Earth–Moon tidal evolution is simulated through three distinct phases, with each phase corresponding to a different ocean model (global and hemispherical oceans) as well as distinct plate tectonic backgrounds since 1 Ga (16). Thus, the F22 model takes the effect of the continental configuration into account, which is absent in the T21 model, and the effect of a dynamically evolving surface geometry in a single reconstructed history, which is different from the D21 model. This may explain the better agreement between the geological findings and the F22 model.

### Staircase Pattern in Earth’s Rotational Deceleration Evolution.

The start of the 650 Ma to 500 Ma deceleration period roughly corresponds to the termination of the Cryogenian glaciations, which may imply that a large proportion of Earth’s surface was affected by ocean inundation and consequently an intensification in tidal dissipation (54–56) (*SI Appendix*, Fig. S12 *B*–*D*). During this period, there was a notable increase in the length of continental arcs and the extent of shallow marine shelves (*SI Appendix*, Fig. S12 *B*–*D*). The extent of shallow marine regions plays a crucial role in the tidal dissipation rate since tidal energy dissipation primarily occurs within these areas (14, 53).

From 500 Ma to 350 Ma, the *k* values derived from geological data show a relatively stable plateau (Fig. 3), which is systematically lower than the F22 model (Fig. 4*A*). The latter signature may be due to our chosen prior distributions for *k* ranges from the W15 model (13). Namely, while the staircase pattern is a robust feature in our geological inferences (Fig. 3 and *SI Appendix*, Fig. S10), the absolute position of this pattern on the precession frequency scale is dependent on the chosen prior distribution. Therefore, the fact that the F22 modeled curve occurs around the upper limit of our prior distribution may explain the slight offset between the model and our findings based on data. During the near-constant *k* value time interval, two *k* estimates (Fig. 4*A*) derived from Zeeden et al. (32) and Zhong et al. (57) are inconsistent or only marginally consistent with the new geological observations and the F22 model. The cyclostratigraphic analysis by Zhong et al. (57) relies solely on the main obliquity component (*k*+*s*_3,_ *s_3_* represents the nodal precession frequency of the Earth) for calculating the *k* value. Their result appears to be inconsistent with the tidal models and other geological estimates (32) (Fig. 4*A*). In order to test the sensitivity of the observed staircase pattern, we compared trends estimated from different data combinations (*SI Appendix*, Fig. S10). By fitting these data combinations, we find that although Zeeden et al. (32) does not have a strong influence on the trend of the 650 Ma to 500 Ma interval, it has a noticeable impact on the fitted trend for the other deceleration period from 350 Ma to 280 Ma (*SI Appendix*, Fig. S10).

The F22 model (16) indicates a change in deceleration from 350 Ma to 280 Ma (Fig. 4). For this time interval, the large uncertainty associated with the new *k* estimate at 375 Ma, coupled with the lack of sufficient geologically derived *k* values from this interval, poses a challenge to robustly determine detail of the trend in Earth’s deceleration evolution. By contrast, the earlier sharp drop at 650 Ma to 500 Ma is clearer than the later one from 350 Ma to 280 Ma (Fig. 3 and *SI Appendix*, Fig. S10). However, taking the *k* estimates by Zeeden et al. (32) into account leads to a changepoint and sharper drop in deceleration from 340 Ma to 245 Ma (Fig. 3 and *SI Appendix*, Fig. S10). Our study provides discrete snapshots of the Earth’s deceleration evolution, which is in good agreement with the F22 model, while a more comprehensive description of this interval requires additional high-quality geological datasets analyzed with improved quantitative methods (e.g., refs. 32 and 58).

### Earth’s Rotational Deceleration Stalled in the Paleozoic (ca. 500 Ma to 350 Ma).

The notable stalling of the Earth’s deceleration occurs in the Paleozoic from ca. 500 Ma to 350 Ma (Fig. 3). Variations in the Earth’s tidal dissipation (14, 16) and dynamical ellipticity (16, 58–61) are the main drivers of the Earth’s rotational evolution. At zeroth order, corresponding to the absence of planetary perturbations, *k* can be formulated as (6)

[1] $$k=\frac{3}{2}\left[\frac{Gm_{s}}{a_{s}^{3}}+\frac{Gm_{M}}{a_{M}^{3}}\right]\frac{H}{\omega }\text{cos}\varepsilon ,$$

where *G* is the gravitational constant, $m_{s}$ is the mass of the Sun, $a_{s}$ is the Earth’s semimajor axis, $m_{M}$ is the mass of the Moon, $a_{M}$ is the lunar semimajor axis, ω is the rotational velocity of the Earth, *H* is its dynamical ellipticity, and *ε* is the Earth’s obliquity. As such, variations in the precession frequency are predominantly controlled by variations in the ratio *H*/ω and $a_{M}$. Consequently, a static Paleozoic *k* can be achieved in one of two ways: either the three quantities (i.e., *H,* ω, $a_{M}$) were nearly constant during the Paleozoic, or they varied with opposing contributions rendering constant *k*. *H* can be decomposed as

[2] $$H=H^{f}+\delta H,$$

where $H^{f}$ denotes the fluid limit of the Earth’s dynamical ellipticity corresponding to its hydrostatic equilibrium (59–63) and δH is the departure from this equilibrium. The evolution of the former component is controlled by the varying Earth rotation rate via (64)

[3] $$H^{f}\approx K_{2}^{T,f}\frac{\omega^{2}R_{E}^{5}}{3GC},$$

where $K_{2}^{T,f}\approx 0.97$ is the fluid limit of the tidal Love number, *R_E_* is the Earth’s mean radius, and *C* is the polar moment of inertia. Ellipticity variations deviating from this hydrostatic figure are driven by surface and internal mass redistributions caused by glacial cycles, isostatic adjustment, and mantle convection (61–68). Tidal dissipation governs the evolution of *k* by evolving $a_{M}$, ω, and $H^{f}$, while the other mass redistribution mechanisms contribute to *k* via δH. Estimates of the effects of these mechanisms have now been explored to some extent for the Cenozoic (59, 61–65, 68), and to a lesser degree for earlier eras (61).

The period of static *k* from 500 Ma to 350 Ma appears to coincide with continental drift toward the South Pole and substantial glacial accumulation in that region. There are roughly two periods of major glaciation overlapping with this time interval: the Late Ordovician (Hirnantian, ca. 445 Ma) glaciation and the Late Paleozoic Ice Age (LPIA, ca. 350 Ma to 280 Ma). The main intense phase of glaciation at the end of the Ordovician was probably short-lived (<5 My) (69). Although this ice age strongly influenced the Earth’s climate, is associated with major carbon cycle perturbations and linked to the Late Ordovician mass extinction (70, 71), it seems unlikely to be the main cause for the long-lasting (ca. 150 My) stasis in *k* due to its shortness in duration, and its occurrence in the middle of the stasis period (Fig. 3). However, on shorter timescales, the Hirnantian glaciation may have exerted an influence on Earth’s rotational deceleration. In contrast, the LPIA was much longer in duration and may thus have had a longer-term effect (e.g., ref. 72).

**Fig. 5.**
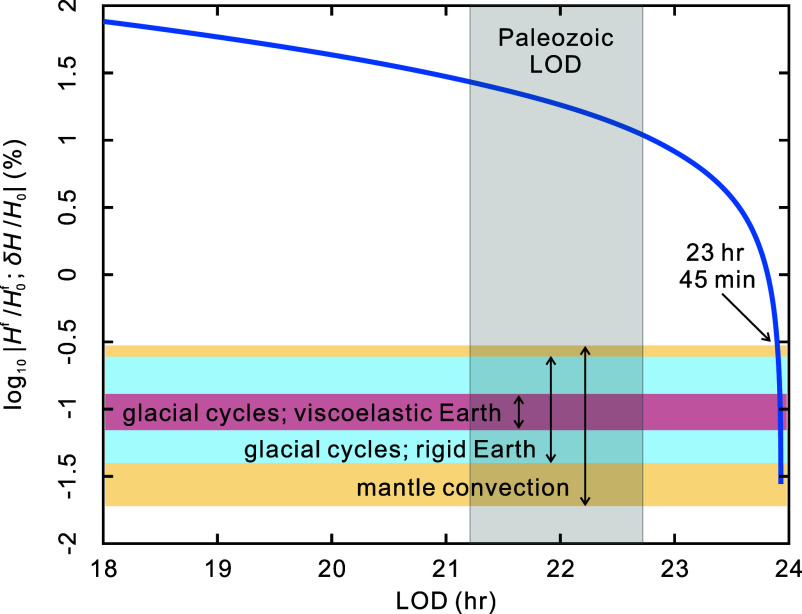
The different geophysical contributions to variations in dynamical ellipticity. Relative variations [${(H}^{f}-H_{0}^{f})/H_{0}^{f}$] in the hydrostatic figure of the Earth, $H^{f}$, relative to its present value, $H_{0}^{f}$, as a function of the LOD is plotted as the blue curve, using Eq. **3**. Note that the *y*-axis is labeled on a logarithmic scale with basis 10, so each increment (e.g., from 0 to 1) suggests 10-fold changes. The yellow region marks the estimates of the relative variation in the dynamical ellipticity, δH, due to mantle convection (67). Mantle convection may induce the strongest nonhydrostatic variation in the dynamical ellipticity with a range of perturbation relative to the present within [−0.5%, −1.7%], depending on the Earth’s viscosity profile. The blue region represents the relative variation in the dynamical ellipticity due to glacial cycles on a rigid Earth (64), that is, ignoring the effect of the Earth’s viscoelasticity and isostatic adjustment. The red region represents the relative variation in the dynamical ellipticity due to glacial isostatic adjustment for a realistic viscoelastic Earth (64). Note that the yellow, blue, and red regions overlap. The red region is thus narrower than the blue region by virtue of the viscoelastic adjustment of the Earth to reestablish its equilibrium figure.

Here, we explore the effect of surface mass redistribution caused by developing ice sheets on the reduced *H* and its potential to offset the tidal braking of ω and preserve the ratio *H*/ω, thus explaining the static *k*. In Fig. 5, we show the relative variations in *H* due to variations in $H^{f}$ (the blue curve) and δH (the colored regions, corresponding to different geophysical mechanisms). In the absence of precise Paleozoic simulations of glacial loading and mantle convection, we consider available estimates for the Cenozoic (61, 64, 67), and use the maximum possible amplitudes of these effects to construct the shaded regions shown in Fig. 5. Fig. 5 shows that surface and internal mass redistributions dominate variations in present-day dynamical ellipticity. However, the contribution of tidal friction to the hydrostatic figure grows significantly for a more rapidly rotating Earth and dominates over the other contributions for LOD shorter than 23 h 45 min (Fig. 5). This dominance grows to a difference in the contributions of almost two orders of magnitude in the Paleozoic (Fig. 5). Therefore, variation of Paleozoic dynamical ellipticity was predominantly controlled by the tidally varying hydrostatic figure, $H^{f}$ (Eq. **3**), rather than by surface mass redistribution. That is, the effect of the tidally controlled rotation rate on $H^{f}$ is more important than the contribution of δH. Specifically, for the effect of Paleozoic glaciation to be comparable to that of tidal friction, we would need an ice sheet that is 70 times more massive than the combined ice sheets of the Last Glacial Maximum (20,000 y ago), concentrated either at the poles or at the equator to maximize the ellipticity variation.

Assuming that such a “super ice sheet” existed, however, the associated surface mass redistribution would largely be compensated by the viscoelastic response of the Earth, which attempts to reestablish hydrostatic equilibrium via isostatic adjustment of the dynamical figure (61, 64, 65). Moreover, this super ice sheet would also need to last for about 150 My to maintain the stasis in *k.* Based on our current state of knowledge, there is no evidence to indicate that such a super ice age has occurred at any time in Earth history. We also observe that main LPIA development occurred only toward the end of the static trend in *k* (Fig. 3), which further suggests that the Paleozoic ice sheet was not the main cause for stasis in *k*. Therefore, we can presume that the stalling of *k* in the Paleozoic observed in Fig. 3 was due to a nearly constant Earth rotation rate and lunar semimajor axis. The latter can be achieved by attenuated tidal dissipation in the Earth’s paleo-oceans, driven by the continent-ocean configuration and the specific LOD at that time (16).

### Earth’s Rotational Deceleration and Relations to Earth’s Environment.

The global tectonic and climatic evolution of the Earth have influenced changes in Earth’s tidal dissipation and dynamic ellipticity (59–68), which ultimately control the Earth’s rotational deceleration evolution. Thus, one may anticipate a relationship between Earth’s rotation and specific geological processes, with various potential connections having been proposed (*SI Appendix*, Figs. S12–S14; refs. 2, 53, 61, and 73–78). For example, daylength changes may influence the distribution of solar energy and temperature gradients, potentially impacting weather systems and atmospheric dynamics (78). Previous studies have also demonstrated that changes in daylength have impacted the Earth’s diurnal illumination dynamics, thereby influencing net oxygen production before and after the Great Oxygenation Event (GOE) (77). In our results, the first high slope of LOD overlaps with the Neoproterozoic Oxygenation Event (NOE) (46, 79) and the Cambrian Explosion (80) (*SI Appendix*, Fig. S13). The second high slope of LOD aligns with the Paleozoic Oxygenation Event (POE) (47–49) and the late Carboniferous to early Permian biodiversification events (*SI Appendix*, Fig. S13). We acknowledge that the precise timings of the NOE and POE are largely inferred from modeling and have not yet been empirically interrogated, and are therefore to be interpreted with care. Nonetheless, the change in daylength may play a triggering mechanism role in these two oxygenation events, akin to the GOE (77). Moreover, the two high slopes of change in LOD are caused by enhanced tidal dissipation (16). This implies that during these two time intervals, high tidal energy dissipation states and enhanced tidal mixing conditions were established in the ocean, which would be beneficial for the formation of oxidative marine environments (81, 82) and favor the survival and evolution of marine ecosystems (83). Thus, there is a potential connection between changes in LOD and the evolution of ocean circulation and marine ecosystems (53, 77, 81–83).

## Materials and Methods

### Evaluation and Screening of Published Cyclostratigraphic Datasets.

For this study, we compiled a suite of cyclostratigraphic time series from published papers (*SI Appendix*, Table S1). First, these cyclostratigraphic data are leveraged to estimate the SR based on the age models provided in the original publications (*SI Appendix*, Table S2) to establish a prior hypothesis for the SR range used in the TimeOpt and TimeOptMCMC analysis. Second, the evolutionary Fast Fourier Transform (eFFT) analysis is applied to identify the most significant and stable interval of astronomical signals, with particular emphasis on climatic precession and orbital eccentricity signals. Third, for the most promising selection of cases based on the eFFT analyses, the TimeOpt method is employed to investigate the amplitude modulation relationship between climatic precession and orbital eccentricity signals and to determine the optimal SR and duration within the chosen interval (Fig. 1 and *SI Appendix*, Figs. S1–S7). Finally, the decision to perform the TimeOptMCMC analysis was based on the *r^2^_opt_* and *P-*values obtained from TimeOpt and TimeOptSim (*SI Appendix*, Table S1). We identified eight high-fidelity datasets from the literature (excluding those analyzed by refs. 30 and 35) that were suitable for TimeOptMCMC analysis (*SI Appendix*, Table S1 and *R scripts*). These datasets are as follows:

(a) The Guandao section was deposited in a deep-marine environment during the latest Permian through the early Middle Triassic (37). A ca. 260 m gamma ray (GR) dataset was retrieved from this section for cyclostratigraphic analysis (37). Variations in GR relate to the terrestrial input and marine productivity, which was controlled by astronomical forcing (37). Based on the Conodont biostratigraphy and magnetostratigraphy, the Guandao section has an average sedimentation rate ranging from 5.5 cm/ky to 7 cm/ky (37). We chose the 10 m to 72 m interval at ca. 245 Ma to run the TimeOptMCMC simulation.

(b) The Permian Lucaogou Formation (ca. 290 Ma) was developed in a deep- lacustrine environment, and consists mainly of shale facies with thin beds of dolomitic siltstone as a minor lithology. The downhole logging natural GR data show strong variations associated with orbital forcing (33). According to the biostratigraphy, detrital U-Pb ages, and varve chronology, the Lucaogou formation has an average sedimentation rate around 10 cm/ky (33). We chose the 3,650 m to 3,770 m interval to perform the TimeOptMCMC analysis.

(c) The H-32 drilling core was drilled at a carbonate platform margin and recorded a positive δ^13^C excursion associated with the Frasnian–Famennian (F–F) boundary during the Upper Devonian (38). The magnetic susceptibility (MS) data revealed quasi-periodic signals at eccentricity, obliquity, and precession bands (38). The H-32 prior age model was established by visually correlating distinct features in MS and carbon isotope geochemistry, yielding a sedimentation rate ranging from 0.7 cm/ky to 1 cm/ky (38). Although the precession band signals are not obvious (*SI Appendix*, Fig. S2), we still chose the 1.76 m to 9 m interval (ca. 375 Ma) for TimeOptMCMC analysis. This choice was necessitated by the absence of any other available cyclostratigraphic dataset capable of reconstructing Earth’s rotation rate within the time frame spanning from 290 Ma to 400 Ma (Fig. 3). Consequently, it may play a crucial role in constraining potential trends in Earth’s rotation deceleration trajectory during this period, although the reconstructed *k* value features a relatively high uncertainty (*SI Appendix*, Table S1).

(d) The Požár-CS limestone section deposited in a deep-marine environment and has a thickness of 118 m, covering the Lochkov and Praha Formations. MS was measured in this section by Da Silva et al. (39). Cyclostratigraphic analysis of the MS data revealed Milankovitch signals (39). The Požár-CS limestone section has a relative wide range in sedimentation rate (0.1 cm/ky to 3 cm/ky) based on the magnetostratigraphy (39), but for our chosen data interval (106.7 m to 114 m, ca. 410 Ma), the sedimentation rate was lower than average and around 0.7 cm/ky (39).

(e) At Anticosti Island, Canada, a well-preserved Upper Ordovician reference section was deposited within a structural embayment situated along the eastern margin of Laurentia. The Vauréal Formation, deposited in a mid- to outer-shelf environment primarily comprises interbedded micrite, calcarenite, and marl, exhibiting lithological alternations that have been interpreted to be forced by astronomical cycles (40). Potassium (K%) was measured to track the multimeter cycles of carbonate versus clay lithology (40). Based on the biostratigraphy (conodont, graptolite, and chitinozoan) and chemostratigraphy (^87^Sr/^86^Sr and δ^13^C_carb_ isotope), the entire ca.1,100 m thick Vauréal Formation represents a few million years (40). We chose the 550 m to 900 m interval (ca. 448 Ma) for TimeOptMCMC analysis.

(f) The Liangjiashan section, located along the margin of the North China Block, represents the deposition of shallow marine carbonate during the Early Ordovician. A set of geochemical data points derived from X-ray fluorescence (XRF) analysis was obtained at the Liangjiashan section (41). These data include elemental contents of Ti, Si, Fe, and Ca. Milankovitch cycles were identified in the Liangjiashan section by analyzing the Ca% (41). Integrating the conodont biostratigraphy and chemostratigraphy of the Liangjiashan section, yielding the sedimentation rate ranging from ca. 0.1 cm/ky to ca. 1.8 cm/ky (41). We chose the 45 m to 62 m interval (ca. 470 Ma) for TimeOptMCMC analysis.

(g) The Alum Shale Formation was deposited in the deeper parts of an epicontinental sea covering the (current) western part of Balticais and is primarily composed of laminated, organic-rich mudstone characterized by a substantial presence of pyrite. Elemental abundances were retrieved using high-resolution core scanning XRF analysis (42). Based on the biostratigraphy and carbon isotope stratigraphy, indicating the Alum Shale Formation has a very low sedimentation rate ranging from ca. 0.14 cm/ky to ca. 0.45 cm/ky (42). By analyzing the S% composition, a floating timescale calibrated to the stable 405 ky eccentricity cycle was established for an approximately 8.7 Ma interval spanning the Miaolingian–Furongian boundary (42). We chose the 83 m to 85.5 m (ca. 493 Ma) interval for TimeOptMCMC analysis.

(h) The Doushantuo Formation was deposited on the inner shelf of the Ediacaran Yangtze Platform at the Zhengjiatang section. Within this section, high-resolution MS series were obtained from the stratigraphic interval containing the Shuram carbon isotope excursion (CIE) (43). Based on the Re–Os ages and carbon isotope stratigraphy, the Doushantuo Formation has an average sedimentation rate around 0.7 cm/ky to 0.8 cm/ky (43). Power spectral analyses conducted on the MS series of the carbonate rocks demonstrate periodicities that align closely with the Milankovitch cycles at ca. 570 Ma (43). We chose the 26 m to 33 m interval to perform TimeOptMCMC analysis.

### TimeOpt, TimeOptSim, and TimeOptMCMC Analysis.

Following the approach of ref. 30, all of these selected geological data showing climatic precession and orbital eccentricity periods were first tested using the TimeOpt method to test for an astronomical signal under a relatively wide range of sedimentation rates (SR). The SR priors were based on the interpretations suggested in the original publications (*SI Appendix*, Table S2). TimeOpt provides the *r^2^_opt_* to evaluate the optimal SR, testing for spectral distributions, while simultaneously fitting the hypothesized climatic precession envelope with expected orbital eccentricity modulation (84). Monte Carlo simulation (TimeOptSim) with a first-order autoregressive model, is utilized to ascertain the statistical significance (*P-*value) of the observed *r^2^_opt_* value (Fig. 1 and *SI Appendix*, Figs. S1–S7). Statistically significant TimeOpt (*r^2^_opt_*) and TimeOptSim (*P-*value) results (*SI Appendix*, Table S1) are an important prerequisite for running the MCMC optimization. Then, Bayesian inversion of these geological records is constrained by prior distributions for the fundamental frequencies *g*_1_ to *g*_5_, the axis precession frequency *k*, and SR (*SI Appendix*, Table S2). Prior distributions for the fundamental frequencies *g*_1_ to *g*_5_ are based on the full range of variability in the model simulations of Laskar et al. computed over 500 My (6). The prior distribution for the axis precession frequency *k* is derived from the study by Waltham (13)_,_ which provides a relatively wide range of possibility. Importantly, in this study, we note that the different choice of the prior distribution may slightly affect the outcomes of the TimeOptMCMC analysis, but the pattern in variations of our datasets is robust, which is independent from the prior distribution. For different cyclostratigraphic datasets, we have run different number of MCMC chains and samples (*SI Appendix*, Table S1), and then we extracted the after burn-in results of all MCMC chains to calculate the mean value of each parameter with its SD (±1σ). For more detailed information about the TimeOpt and TimeOptMCMC methods refer to ref. 30.

### Change-Point Analysis.

A change point is a sample or time instant at which some statistical property (for instance: mean value, SD, trend) of a signal change abruptly (45). The MATLAB function “findchangepts” can be used to detect the change points in a time series. We have employed this function to estimate the “linear” statistic properties of the cyclostratigraphically derived *k* values time span from 700 Ma to 200 Ma (Fig. 3). To display the abrupt changes on these data, we plot the linear regression lines of different data groups and calculate the mean slope of all regression lines (Fig. 3). In summary, our statistical analysis suggests the presence of two discernible change points/intervals (ca. 650 Ma to 480 Ma, ca. 350 Ma to 280 Ma) based on these data (Fig. 3 and *SI Appendix*, Fig. S10).

## Supplementary Material

Appendix 01 (PDF)

## Data Availability

Previously published data were used for this work (refs. 32 and 37–43). All other data are included in the manuscript and/or *SI Appendix*.
