# Supplementary material for: Geological evidence reveals a staircase pattern in Earth’s rotational deceleration evolution
Source: Proc Natl Acad Sci U S A. 2024 Aug 6;121(33):e2317051121. doi: 10.1073/pnas.2317051121 (PMC11331090; doi:10.1073/pnas.2317051121)
Supplement: Supplementary file 1 — Appendix 01 (PDF) [file pnas.2317051121.sapp.pdf]

PNAS

Supporting Information for:

Geological evidence reveals a staircase pattern in Earth's rotational deceleration evolution

He Huang[1,2,3], Chao Ma[1,2,] *, Jacques Laskar[3], Matthias Sinnesael[3,4], Mohammad Farhat[3], Nam H. Hoang[3], Yuan Gao[5], Christian Zeeden[6], Hanting Zhong[1,2], Mingcai Hou[1,2], Chengshan Wang[5]

[1] State Key Laboratory of Oil and Gas Reservoir Geology and Exploitation, Institute of Sedimentary Geology, Chengdu University of Technology, Chengdu 610059, China

[2] Key Laboratory of Deep-time Geography and Environment Reconstruction and Applications of Ministry of Natural Resources, Chengdu University of Technology, Chengdu 610059, China

[3] IMCCE, CNRS, Observatoire de Paris, PSL University, Sorbonne Université, 75014, Paris, France

[4] Department of Geology, School of Natural Sciences, Trinity College Dublin, The University of Dublin, College Green, Dublin 02, Ireland

[5] State Key Laboratory of Biogeology and Environmental Geology, China University of Geosciences (Beijing), Beijing 100083, China

[6] LIAG-Leibniz Institute for Applied Geophysics, Stilleweg 2, 30655 Hannover, Germany

Corresponding author: Chao Ma

Email: machao@cdut.edu.cn

This PDF file includes:

Tables S1 to S3

Figures S1 to S14

Supplementary R scripts

SI References

Supplementary Tables

Table S1. Detailed information of the geological data used in this study. We provided some of the key parameters for running the TimeOpt, TimeOptSim and TimeOptMCMC analysis.

Stratigraphic interval	Time (Ma)	Formation /Location/ Fossil	Proxy	TimeOpt r^2_{opt} value	TimeOptSim p-value	TimeOptMCMC Num. of samples and chains	*k* (arcsec/yr)	±σ (arcsec/yr)	Data Resource
**Today**[*]	0 Ma						50.475838		ref. (1)
**Eocene**[§]	41 Ma	Newfoundland Ridge	Ca/Fe				51.28	0.56	ref. (2)
**Eocene**[§]	55 Ma	Walvis Ridge	a*(red/green)	0.212	<0.005	200,000; 150	51.28	0.52	ref. (3)
**Campanian**[†]	80 Ma	Rudist Shell	XRF				52.58	0.44	ref. (4)
**Anisian**	**245 Ma**	**Guandao**	**GR**	**0.207**	**0.012**	**200,000; 100**	**56.70**	**2.26**	**ref. (5)**
**Wuchiapingian**[§]	259 Ma	Wujiaping	ARM	0.246	<0.005	600,000; 50	55.86	1.30	ref. (6)
**Artinskian**	**290 Ma**	**Lucaogou**	**GR**	**0.199**	**0.0115**	**100,000; 150**	**57.06**	**1.36**	**ref. (7)**
**Frasnian**	**375 Ma**	**H-32, Iowa**	**MS**	**0.19**	**0.153**	**100,000; 200**	**59.53**	**3.24**	**ref. (8)**
**Emsian**[§]	~400 Ma		MS				62.61	0.60	ref. (9)
**Pragian**	**410 Ma**	**Požár-CS**	**MS**	**0.162**	**0.023**	**200,000; 150**	**59.72**	**1.89**	**ref. (10)**
**Katian**	**448 Ma**	**Anticosti Island**	**K%**	**0.217**	**0.0165**	**200,000; 100**	**59.02**	**1.63**	**ref. (11)**
**Sandbian**[§]	455 Ma	Pingliang	MS	0.094	0.0295	1,000,000; 30	59.71	1.29	ref. (6)
**Floian**	**470 Ma**	**Liangjiashan**	**Ca%**	**0.121**	**0.0625**	**600,000; 50**	**59.21**	**1.29**	**ref. (12)**
**Jiangshanian**	**493 Ma**	**Alum Shale**	**S%**	**0.184**	**0.0753**	**200,000; 100**	**62.76**	**2.81**	**ref. (13)**
**Cambrian**[§]	500 Ma	Luoyixi section	MS				61.06	0.94	ref. (14)
**Cambrian**[§]	526 Ma	Qiongzhusi	Fe/Al				62.65	1.04	ref. (15)

**Ediacaran**	**570 Ma**	**Doushantuo**	**MS**	**0.189**	**0.1635**	**200,000; 100**	**63.49**	**2.92**	**ref. (16)**
**Cryogenian**§	655 Ma	Datangpo	MS	0.215	0.0355	1,000,000; 30	70.21	2.08	ref. (6)
**Tonian**†	830 Ma	Stromatolites					72.77	/	ref. (4)
**Tonian**†	900 Ma	Tidal laminae					74.90	+8.85/-7.78	ref. (17)
**Mesoproterozoic**§	1400 Ma	Xiamaling	Cu/Al	0.3	<0.005	1,000,000; 50	85.79	1.36	ref. (3)
**Paleoproterozoic**§	2460 Ma	Joffre	Lithological index				108.6	8.5	ref. (18)
**Paleoproterozoic**§	2465 Ma	Dales Gorge	Greyscale	0.087	0.039	1,000,000; 30	105.26	1.35	ref. (6)

*Earth’s rotation rate estimates from ref. (1).

§Earth’s rotation results inferred from cyclostratigraphic analysis from the published articles.

†Earth’s rotation results calculated from the tidalites and/or invertebrate fossil growth cycle from the published articles.

Note: All the errors in this table are one standard deviation (± 1σ), the bold terms in this table are calculated by this study.

GR: gamma ray; ARM: anhysteretic remanent magnetization; MS: magnetic susceptibility.

Table S2. Definition of TimeOptMCMC priors for sedimentation rate, Earth axial precession frequency *k* and secular frequency g_i terms for the datasets used in this study.

Time (Ma)	Sedimentation rate (cm/kyr)	*k* (arcsec/yr)	g_i terms (arcsec/yr)
245	4-7 (ref. 5)	54.5±2.5	
290	2-18 (ref. 7)	55±3	g_1=5.525±0.125
375	0.7-1 (ref. 8)	58±4	g_2=7.455±0.015
410	0.2-1(ref. 10)	58±4	g_3=17.3±0.15
448	10-60 (ref. 11)	59±4	g_4=17.85±0.15
470	0.1-1.8 (ref. 12)	59±5	g_5=4.257455±0.00002
493	0.1-0.4 (ref. 13)	59±5	
570	0.5-0.9 (ref. 16)	60±5	

Note: Prior distributions for the fundamental frequencies g_1 to g_5 are based on the full range of variability in the model simulations of ref. (1) computed over 500 My. The prior distribution for the precession frequency is derived from the study by ref. (19).

Table S3. TimeOptMCMC reconstruction of Earth-Moon system parameters for the eight cyclostratigraphic records.

Time (Ma)	*k* (arcsec/yr)	EMD (1000 km)	LOD (hrs)	Obliquity (°)	SR (cm/kyr)
245	56.7 ± 2.26	374.0 (+3.36/-3.22)	22.6 (+0.46/-0.45)	22.6 (+0.21/-0.21)	6.1 ± 0.14
290	57.1 ± 1.36	373.5 (+1.99/-1.95)	22.5 (+0.28/-0.26)	22.6 (+0.13/-0.12)	10.0 ± 0.20
375	59.5 ± 3.24	367.0 (+4.63/-4.39)	22.1 (+0.62/-0.56)	22.4 (+0.29/-0.27)	0.8 ± 0.04
410	59.7 ± 1.89	367.0 (+2.67/-2.58)	22.1 (+0.36/-0.33)	22.4 (+0.16/-0.16)	0.83 ± 0.011
448	59.0 ± 1.63	370.7 (+2.32/-2.26)	22.2 (+0.31/-0.29)	22.4 (+0.14/-0.14)	47.7 ± 1.51
470	59.2 ± 1.29	370.4 (+1.83/-1.78)	22.2 (+0.24/-0.23)	22.4 (+0.11/-0.11)	1.6 ± 0.02
493	62.8 ± 2.81	365.6 (+3.79/-3.63)	21.5 (+0.48/-0.44)	22.2 (+0.24/-0.22)	0.34 ± 0.008
570	63.5 ± 2.92	364.6 (+3.90/-3.73)	21.4 (+0.49/-0.45)	22.0 (+0.23/-0.23)	0.8 ± 0.02

Note: EMD = Earth-Moon distance; LOD = the length of the day; SR = the optimal sedimentary rate. The reported uncertainties are one standard deviation (1σ).

Supplementary Figures

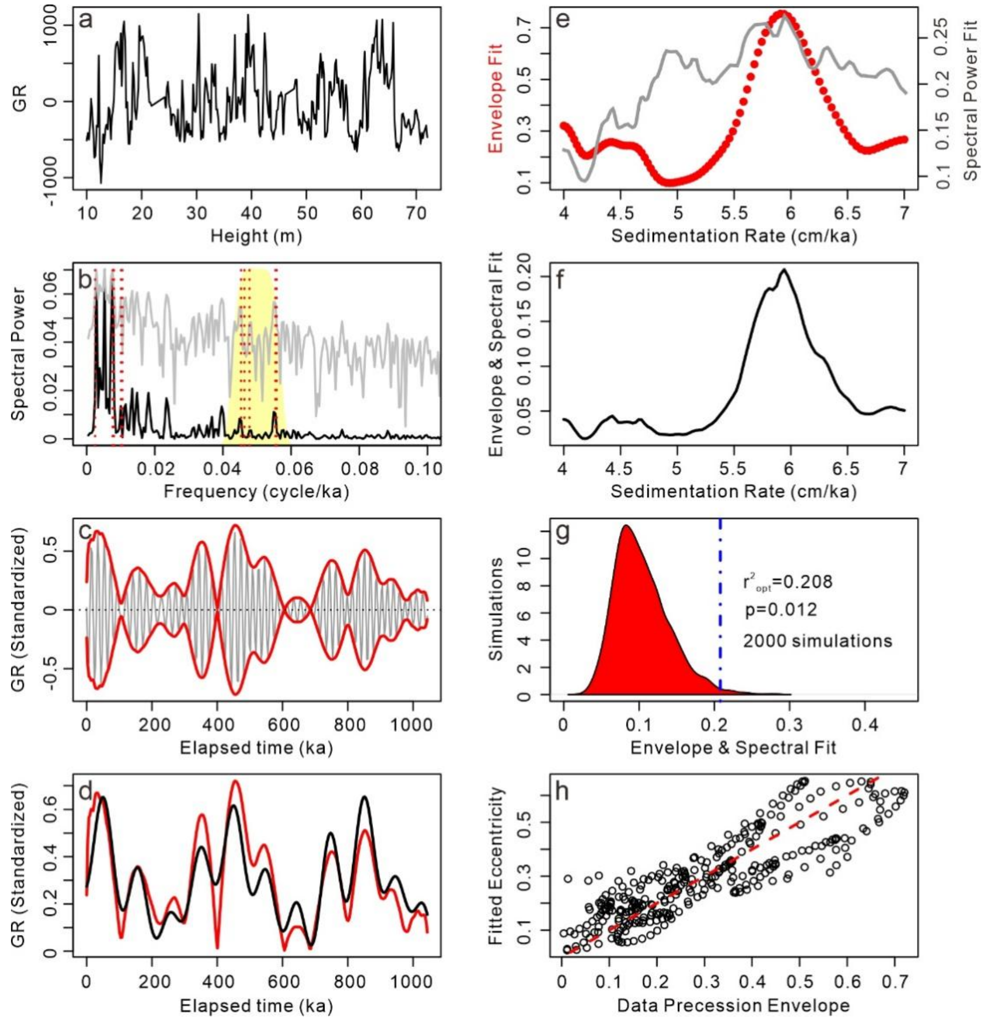

Fig. S1. TimeOpt and TimeOptSim analyses of the GR data from the Guandao section (ca. 245 Ma). (a) The GR data of Guandao section (5). (b) Periodogram for the GR data (black line=linear spectrum; gray line=log spectrum). Yellow shaded region indicates the portion of the spectrum bandpass filtered for evaluation of the precession amplitude envelope. Vertical dashed red lines indicate the eccentricity and climatic precession target frequencies. (c) Extracting the band-passed precession signal (black), and the data amplitude envelope (red) determined via Hilbert transform. (d) Comparison of the data amplitude envelope (red) and the TimeOpt reconstructed eccentricity model (black). (e) Squared Pearson correlation coefficient for the amplitude envelope fit and the spectral power fit as a function of sedimentation rate. (f) Combined envelope and spectral power fit at each evaluated sedimentation rate. (g) Summary of 2000 Monte Carlo simulations with AR1 surrogates. (h) Cross plot of the data amplitude envelope and the TimeOpt-reconstructed eccentricity model in panel "d"; dashed red line is the 1:1 line.

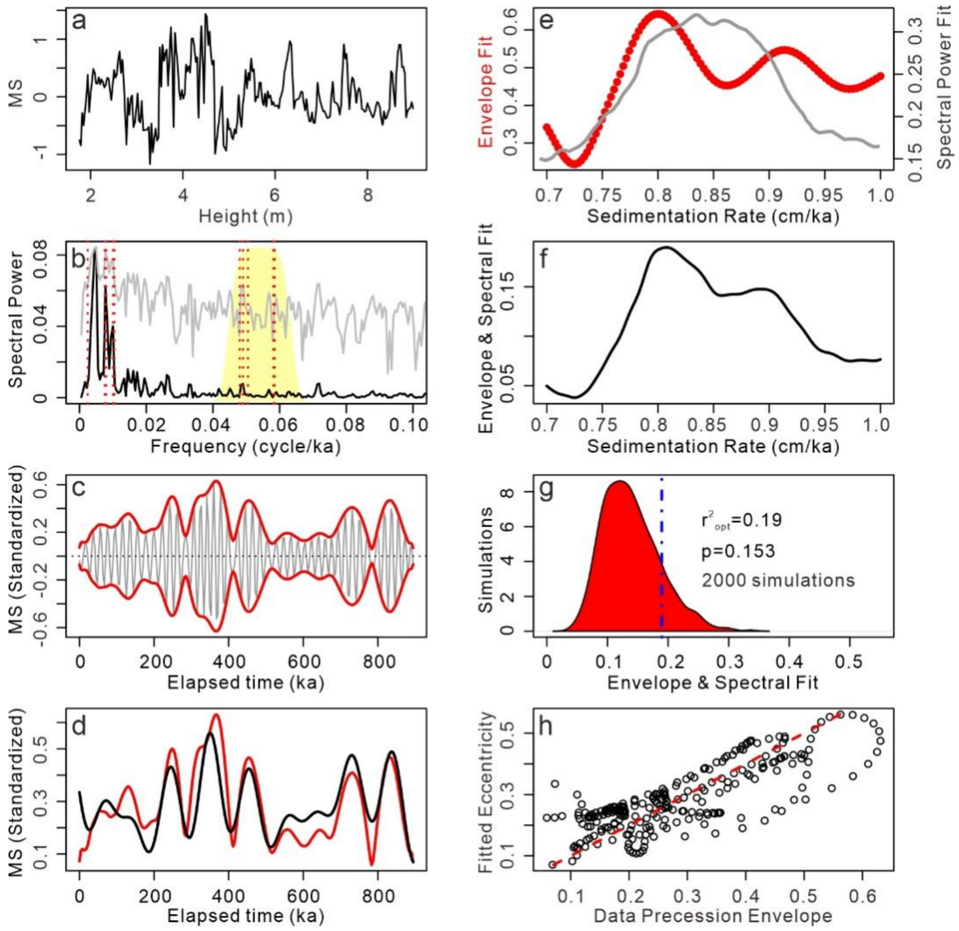

Fig. S2. TimeOpt and TimeOptSim analyses of the MS series from the H-32 core (ca. 375 Ma). (a) The MS data of H-32 core (8). (b) Periodogram for the MS data (black line=linear spectrum; gray line=log spectrum). Yellow shaded region indicates the portion of the spectrum bandpass filtered for evaluation of the precession amplitude envelope. Vertical dashed red lines indicate the eccentricity and climatic precession target frequencies. (c) Extracting the band-passed precession signal (black), and the data amplitude envelope (red) determined via Hilbert transform. (d) Comparison of the data amplitude envelope (red) and the TimeOpt reconstructed eccentricity model (black). (e) Squared Pearson correlation coefficient for the amplitude envelope fit and the spectral power fit as a function of sedimentation rate. (f) Combined envelope and spectral power fit at each evaluated sedimentation rate. (g) Summary of 2000 Monte Carlo simulations with AR1 surrogates. (h) Cross plot of the data amplitude envelope and the TimeOpt-reconstructed eccentricity model in panel “d”; dashed red line is the 1:1 line.

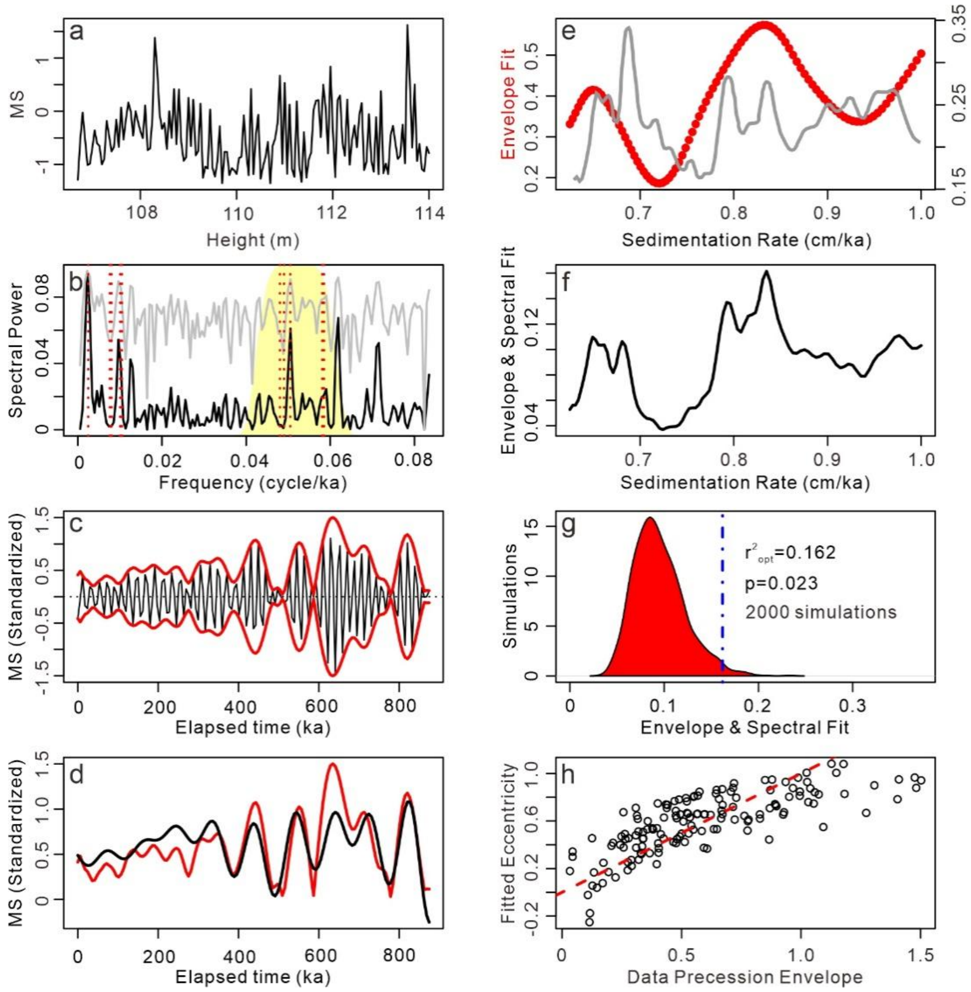

Fig. S3. TimeOpt and TimeOptSim analyses of the MS series from the Požár-CS section (ca. 410 Ma). (a) The MS data of the Požár-CS section (10). (b) Periodogram for the MS data (black line=linear spectrum; gray line=log spectrum). Yellow shaded region indicates the portion of the spectrum bandpass filtered for evaluation of the precession amplitude envelope. Vertical dashed red lines indicate the eccentricity and climatic precession target frequencies. (c) Extracting the band-passed precession signal (black), and the data amplitude envelope (red) determined via Hilbert transform. (d) Comparison of the data amplitude envelope (red) and the TimeOpt reconstructed eccentricity model (black). (e) Squared Pearson correlation coefficient for the amplitude envelope fit and the spectral power fit as a function of sedimentation rate. (f) Combined envelope and spectral power fit at each evaluated sedimentation rate. (g) Summary of 2000 Monte Carlo simulations with AR1 surrogates. (h) Cross plot of the data amplitude envelope and the TimeOpt-reconstructed eccentricity model in panel "d"; dashed red line is the 1:1 line.

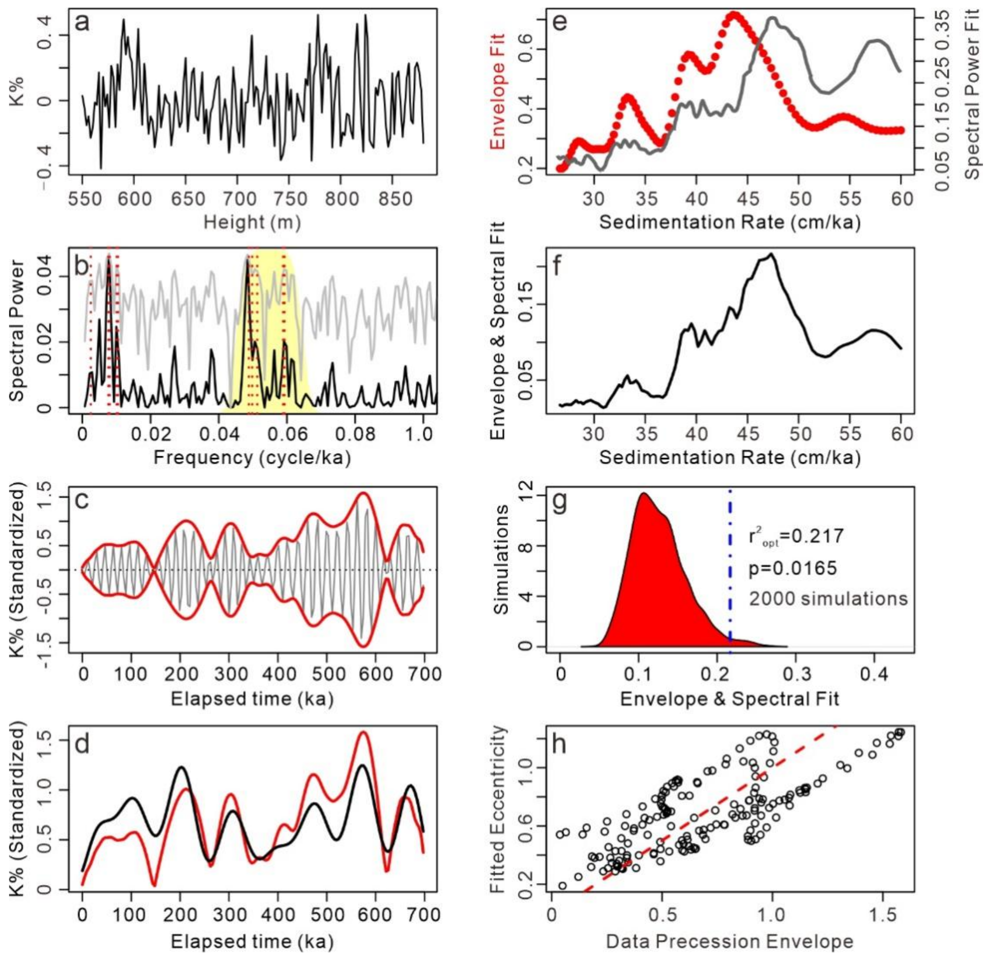

Fig. S4. TimeOpt and TimeOptSim analyses of the K% series from the Upper Ordovician reference section (ca. 448 Ma) in Anticosti Island, Canada. (a) The K% data of the Upper Ordovician reference section (11). (b) Periodogram for the K data (black line=linear spectrum; gray line=log spectrum). Yellow shaded region indicates the portion of the spectrum bandpass filtered for evaluation of the precession amplitude envelope. Vertical dashed red lines indicate the eccentricity and climatic precession target frequencies. (c) Extracting the band-passed precession signal (black), and the data amplitude envelope (red) determined via Hilbert transform. (d) Comparison of the data amplitude envelope (red) and the TimeOpt reconstructed eccentricity model (black). (e) Squared Pearson correlation coefficient for the amplitude envelope fit and the spectral power fit as a function of sedimentation rate. (f) Combined envelope and spectral power fit at each evaluated sedimentation rate. (g) Summary of 2000 Monte Carlo simulations with AR1 surrogates. (h) Cross plot of the data amplitude envelope and the TimeOpt-reconstructed eccentricity model in panel “d”; dashed red line is the 1:1 line.

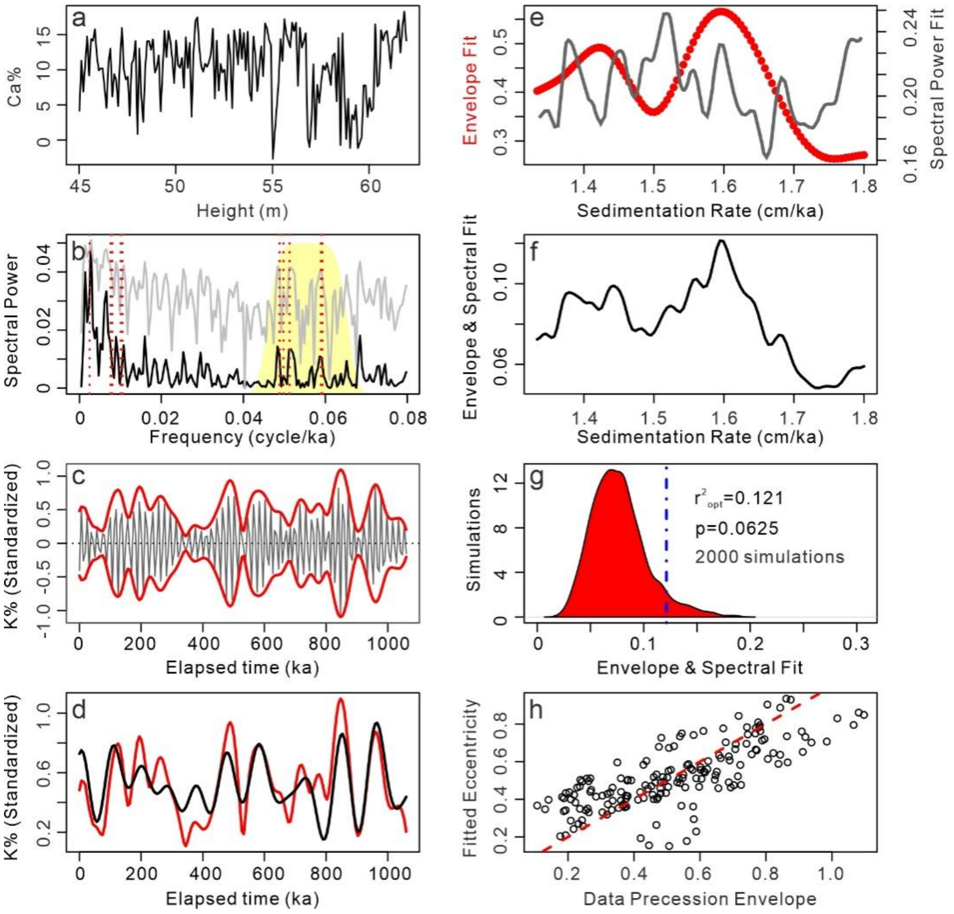

Fig. S5. TimeOpt and TimeOptSim analyses of the Ca% series from the Liangjiashan section (ca. 470 Ma). (a) The Ca% data of the Liangjiashan section (12). (b) Periodogram for the Ca% data (black line=linear spectrum; gray line=log spectrum). Yellow shaded region indicates the portion of the spectrum bandpass filtered for evaluation of the precession amplitude envelope. Vertical dashed red lines indicate the eccentricity and climatic precession target frequencies. (c) Extracting the band-passed precession signal (black), and the data amplitude envelope (red) determined via Hilbert transform. (d) Comparison of the data amplitude envelope (red) and the TimeOpt reconstructed eccentricity model (black). (e) Squared Pearson correlation coefficient for the amplitude envelope fit and the spectral power fit as a function of sedimentation rate. (f) Combined envelope and spectral power fit at each evaluated sedimentation rate. (g) Summary of 2000 Monte Carlo simulations with AR1 surrogates. (h) Cross plot of the data amplitude envelope and the TimeOpt-reconstructed eccentricity model in panel "d"; dashed red line is the 1:1 line.

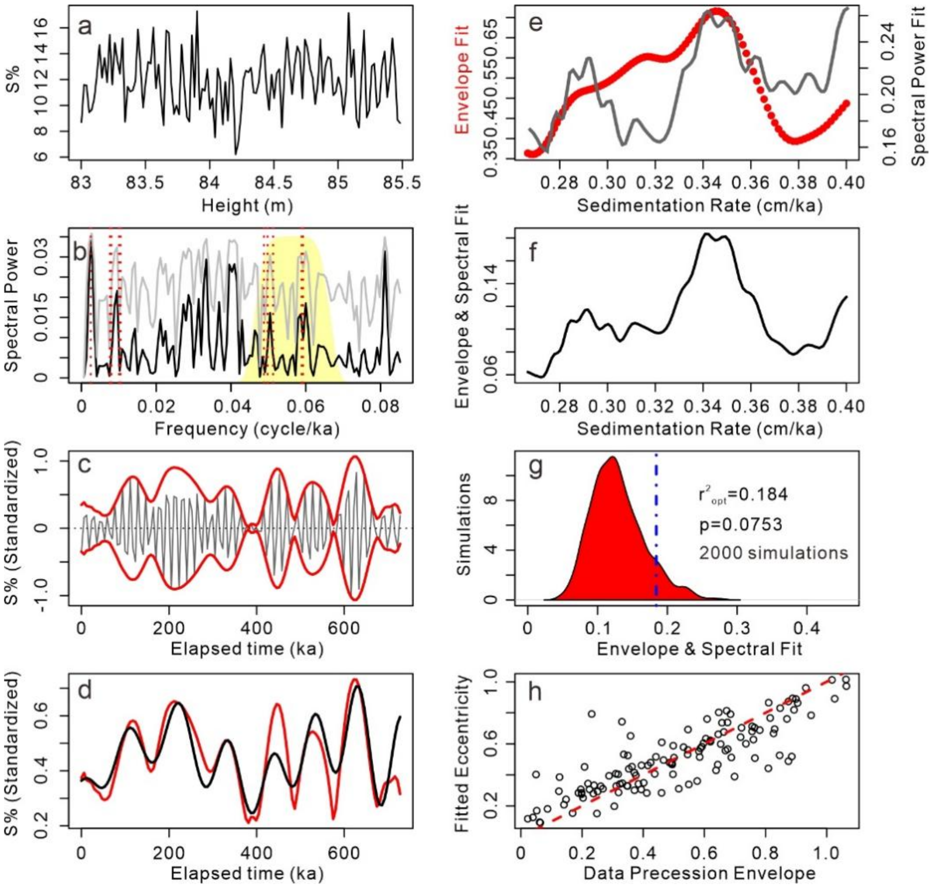

Fig. S6. TimeOpt and TimeOptSim analyses of the S% series from the Alum Shale Formation (ca. 493 Ma). (a) The S% data of the Alum Shale (13). (b) Periodogram for the S% data (black line=linear spectrum; gray line=log spectrum). Yellow shaded region indicates the portion of the spectrum bandpass filtered for evaluation of the precession amplitude envelope. Vertical dashed red lines indicate the eccentricity and climatic precession target frequencies. (c) Extracting the band-passed precession signal (black), and the data amplitude envelope (red) determined via Hilbert transform. (d) Comparison of the data amplitude envelope (red) and the TimeOpt reconstructed eccentricity model (black). (e) Squared Pearson correlation coefficient for the amplitude envelope fit and the spectral power fit as a function of sedimentation rate. (f) Combined envelope and spectral power fit at each evaluated sedimentation rate. (g) Summary of 2000 Monte Carlo simulations with AR1 surrogates. (h) Cross plot of the data amplitude envelope and the TimeOpt-reconstructed eccentricity model in panel “d”; dashed red line is the 1:1 line.

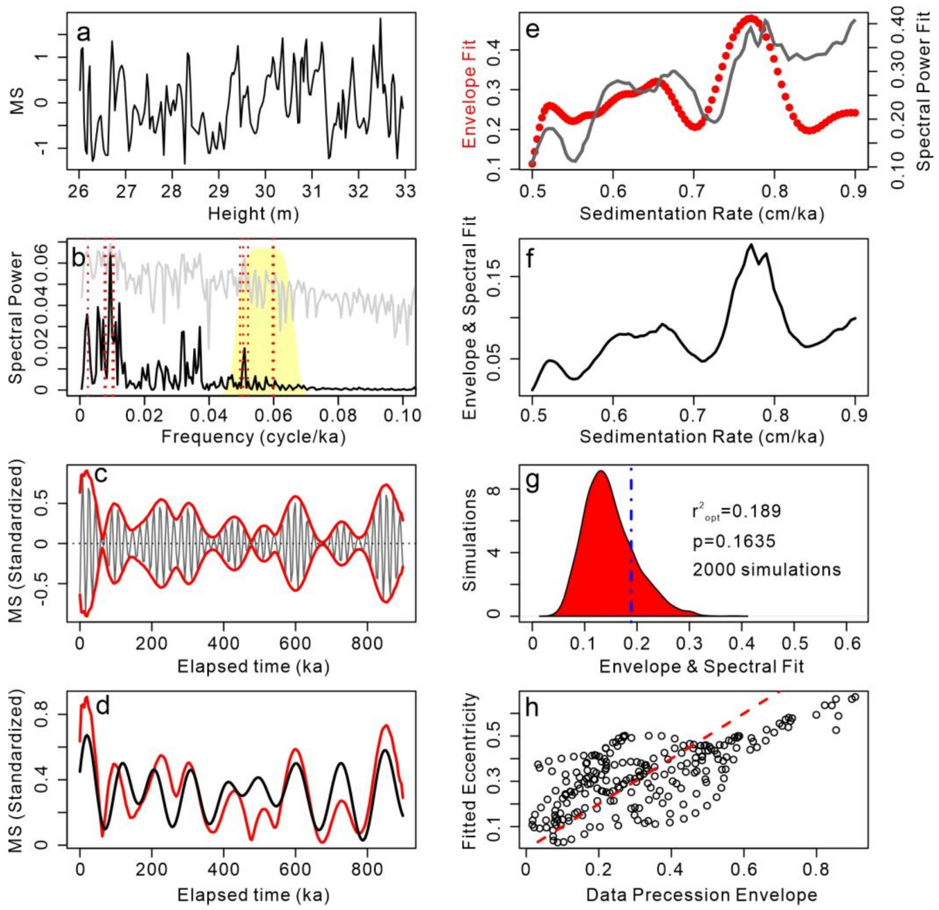

Fig. S7. TimeOpt and TimeOptSim analyses of the MS series from the Doushantuo Formation (ca. 570 Ma). (a) The MS data of the Doushantuo Formation (16). (b) Periodogram for the MS data (black line=linear spectrum; gray line=log spectrum). Yellow shaded region indicates the portion of the spectrum bandpass filtered for evaluation of the precession amplitude envelope. Vertical dashed red lines indicate the eccentricity and climatic precession target frequencies. (c) Extracting the band-passed precession signal (black), and the data amplitude envelope (red) determined via Hilbert transform. (d) Comparison of the data amplitude envelope (red) and the TimeOpt reconstructed eccentricity model (black). (e) Squared Pearson correlation coefficient for the amplitude envelope fit and the spectral power fit as a function of sedimentation rate. (f) Combined envelope and spectral power fit at each evaluated sedimentation rate. (g) Summary of 2000 Monte Carlo simulations with AR1 surrogates. (h) Cross plot of the data amplitude envelope and the TimeOpt-reconstructed eccentricity model in panel "d"; dashed red line is the 1:1 line.

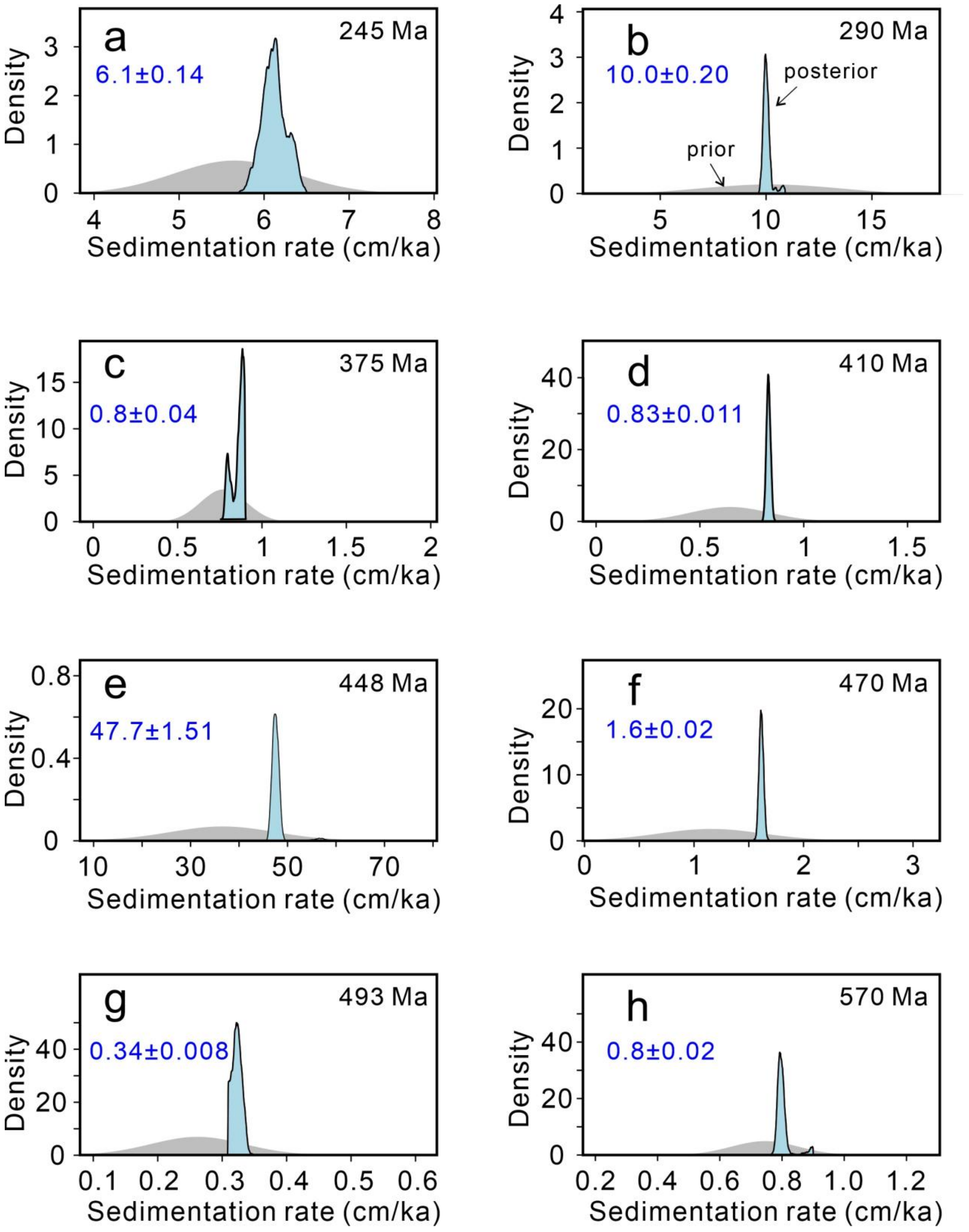

Fig. S8. Prior and posterior distributions of the SR values from TimeOptMCMC analysis of eight cyclostratigraphic data. Shaded grey areas indicate the prior distributions, and blue-shaded histograms indicate the posterior distributions obtained by the Markov-Chain Monte Carlo sampling.

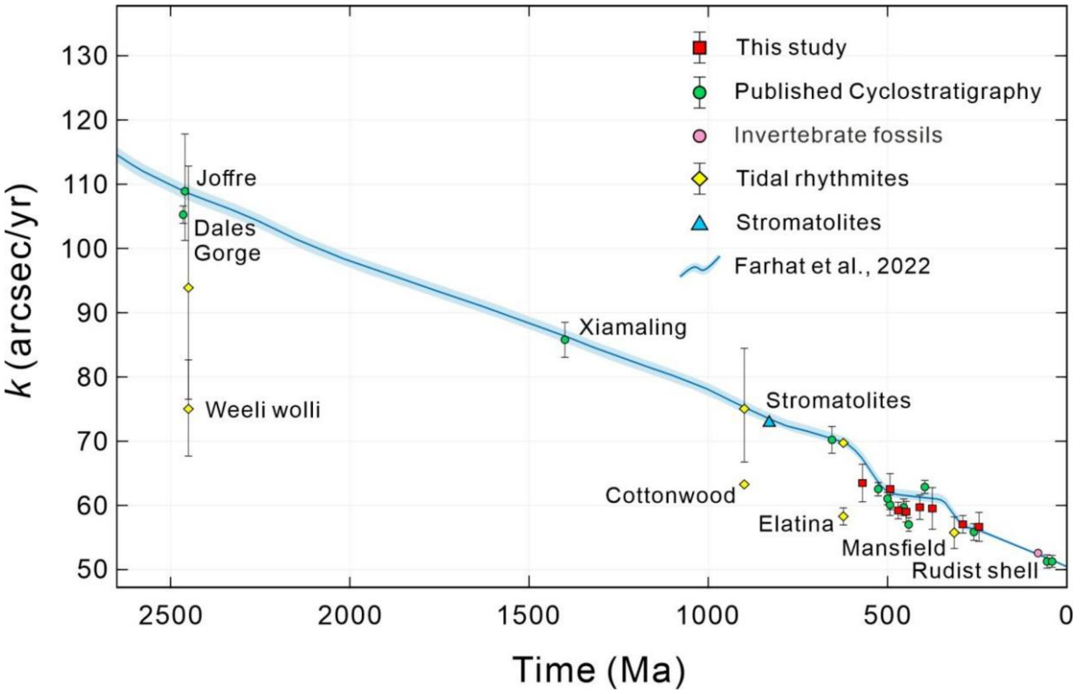

Fig. S9. Geologically derived *k* values (Table S1) and the F22 tidal model (20). The red square points with error bars are from this study, the green circle points with error bars are from published cyclostratigraphic articles, the purple and yellow data points originated from the invertebrate fossils and tidal rhythmites, the blue triangle represents stromatolites, respectively.

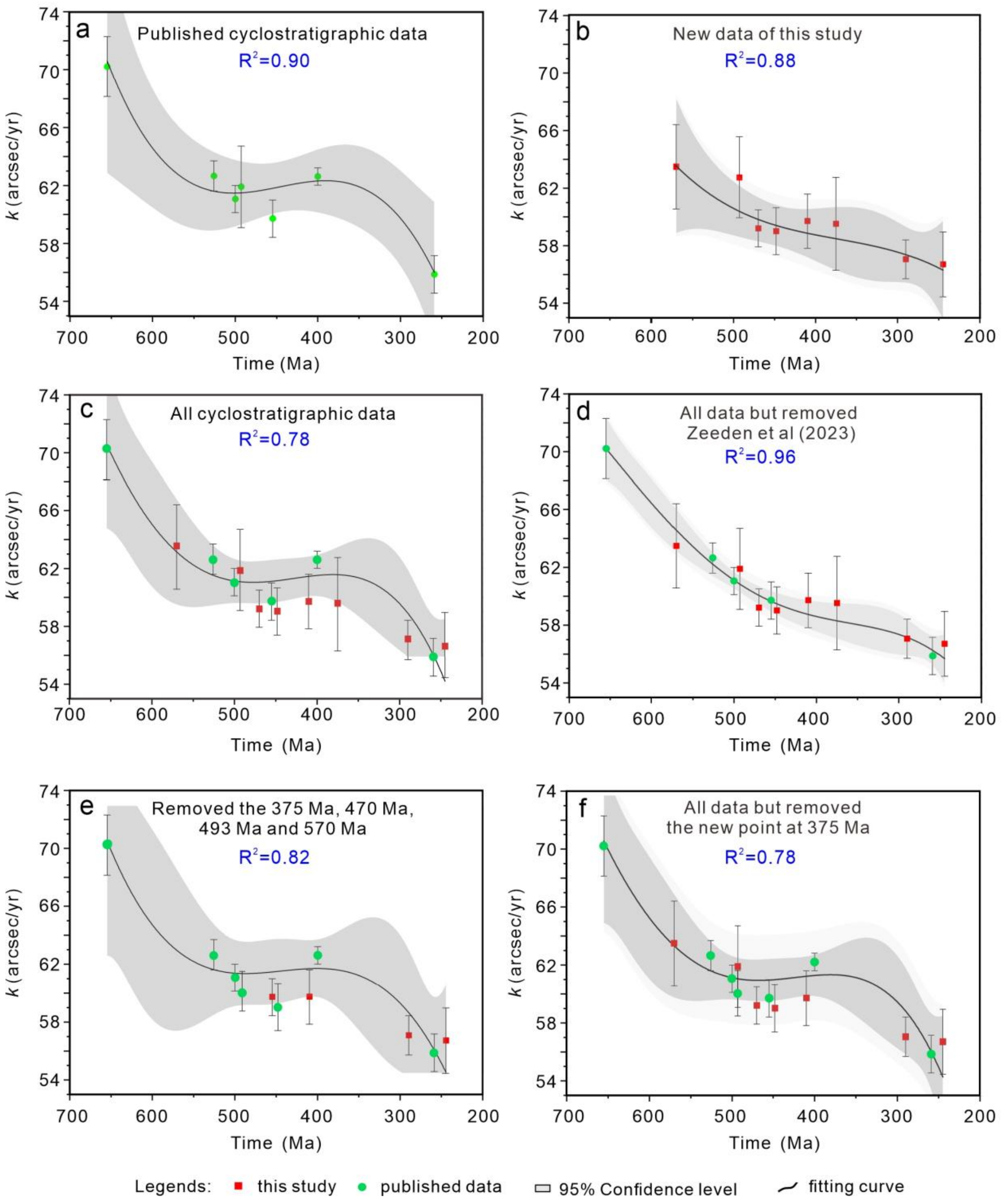

Fig. S10. Fourth-order polynomial fitting different combinations of reconstructed *k* values from 700 Ma to 200 Ma. (a) Fitting the already published cyclostratigraphic data. (b) Fitting only the new cyclostratigraphic data from this study. (c) Fitting all of the cyclostratigraphic data. (d) Fitting all of the cyclostratigraphic data but remove Zeeden et al. (2023). (e) Removed the geologically derived *k* data at 375 Ma, 470 Ma, 493 Ma and 570 Ma, and then fitting the rest of cyclostratigraphic data. (f) Removing only the 375 Ma data point, and then fitting the rest of data.

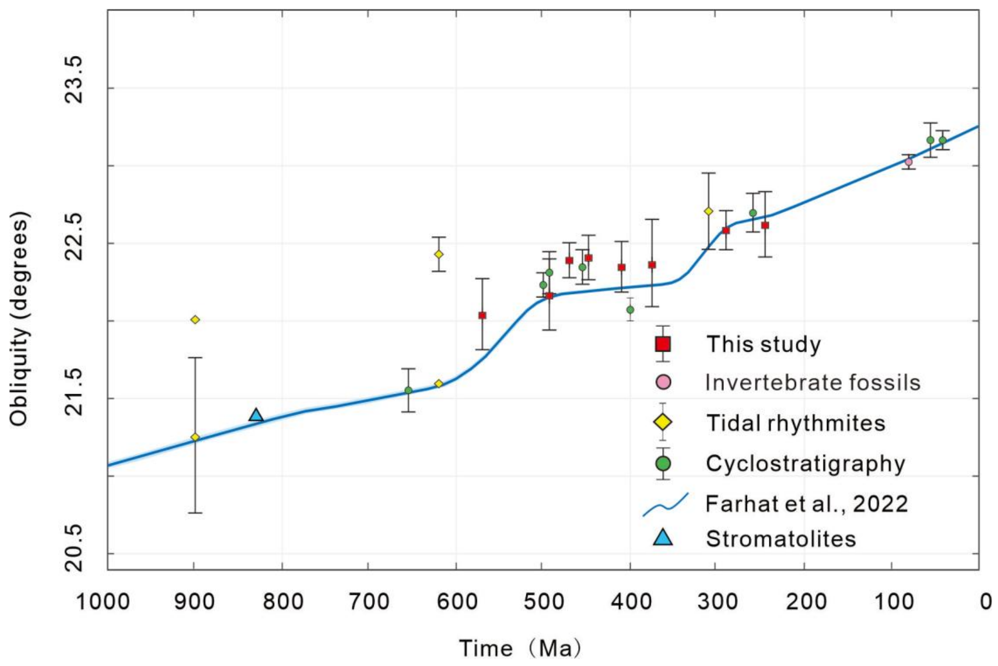

Fig. S11. Comparison of the reconstructed angle of obliquity with the F22 model (20). The red square points with error bars are from this study, the green circle points with error bars are from published cyclostratigraphic articles, the purple and yellow data points originated from the invertebrate fossils and tidal rhythmites, and the blue triangle represents stromatolites, respectively.

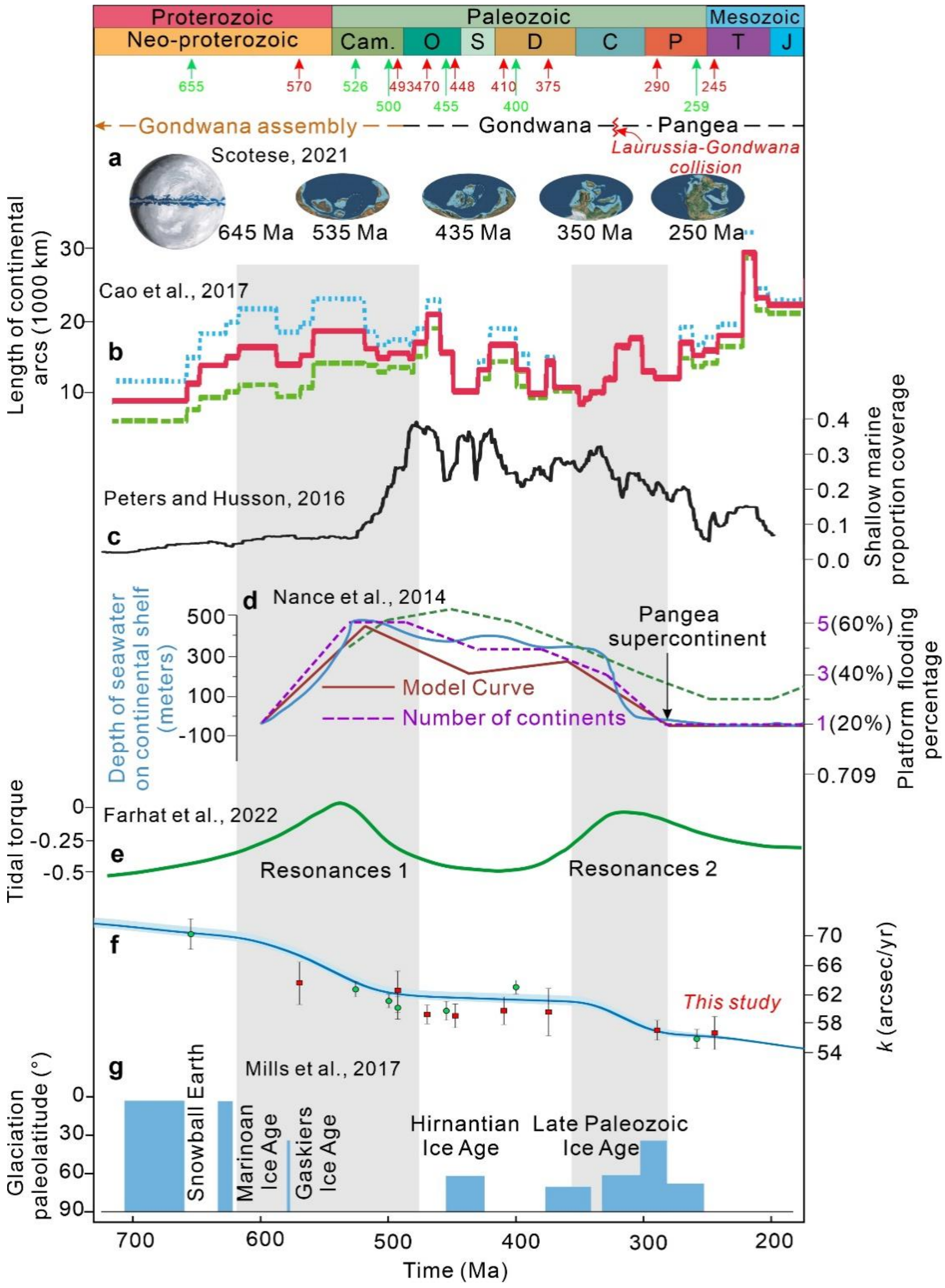

Fig. S12. Comparison between the evolution of Earth's axial precession frequency (k) and the trends of multiple tectonic and environmental records. (a) Paleogeographic maps of the Earth (21). (b) Continental arc length in the past 750 Ma (22). Dotted blue, dashed green, and solid red curves are the maximum, minimum, and average length estimates, respectively. (c) Shallow marine proportion coverage curve (23). (d) Depth of seawater on continental shelf, the degree of platform flooding and the number of continents from the past ~600 Ma to ~190 Ma (24). (e) Simulated tidal torque and normalized its absolute strength to present value (20). (f) Estimated k from geological archives, the blue curve represents the F22 tidal model (20). (g) Paleolatitude of glaciations throughout the Neoproterozoic to Paleozoic (25).

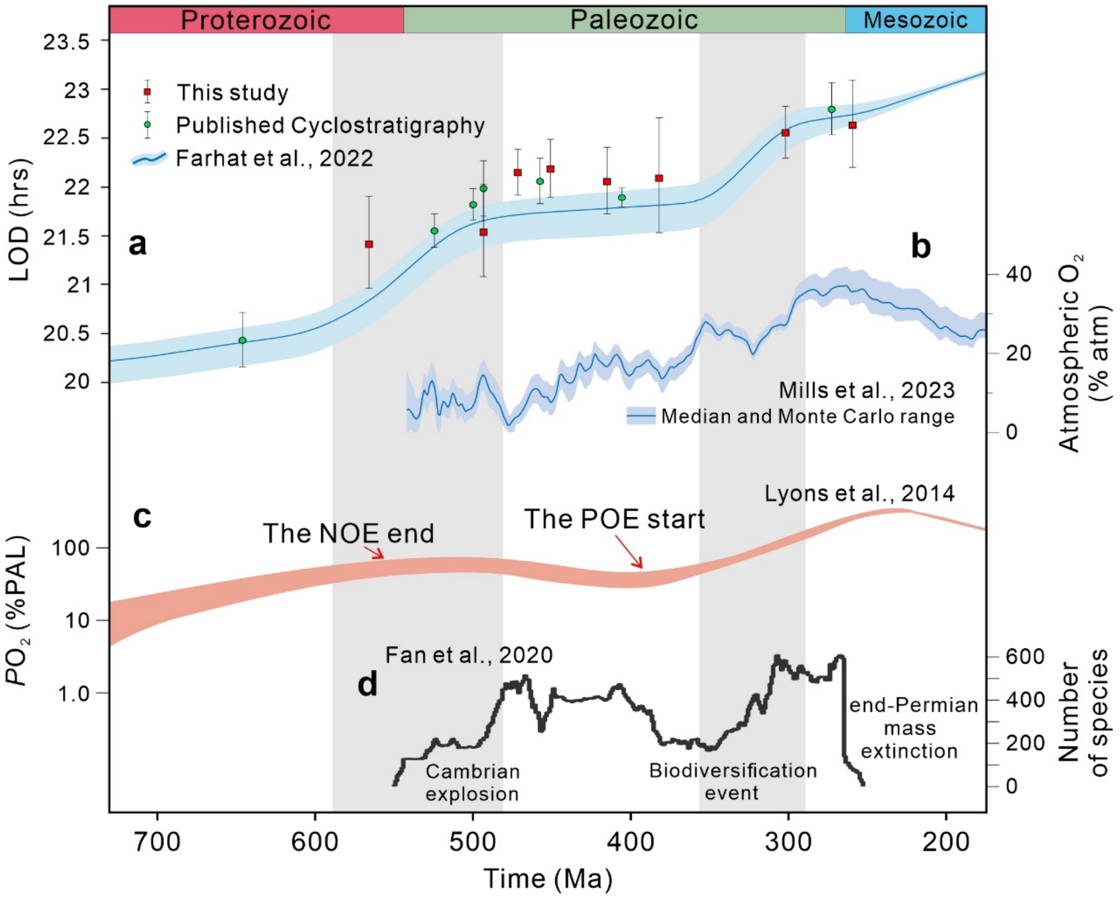

Fig. S13. Comparison between the evolution of LOD and the trends of atmospheric oxygen concentration and species abundance curves. (a) The estimated LOD from geological archives, the blue curve represents the F22 tidal model (20). (b) Model results of the evolution of Earth's atmospheric oxygen content (26). (c) The evolution of Earth's atmospheric oxygen content from Neoproterozoic to Mesozoic Eras (27). (d) The species diversity from Cambrian to Triassic (28).

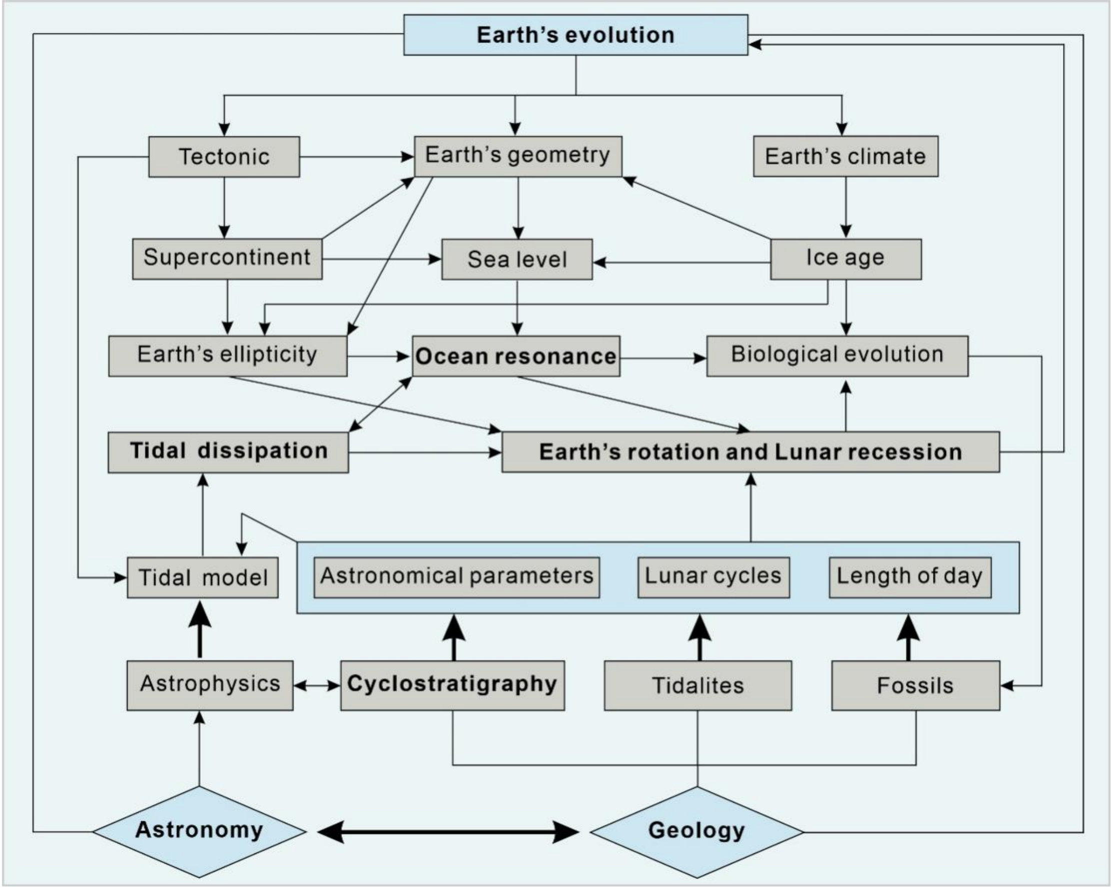

Fig. S14. Schematic of possible cause-and-effect relationships between the Earth's rotational dynamics and geological processes. In this framework, the variations of the Earth-Moon tidal dissipation and Earth dynamic ellipticity dynamic are two main factors that influence Earth's rotational deceleration. Understanding these connections requires interdisciplinary research combining astrophysics, geophysics, geology, climatology, and other relevant fields. Additionally, international collaborations are necessary to solve these complex issues.

Supplementary R scripts (Scripted by He Huang, 2021-2024).

The R Scripts for TimeOpt and TimeOptMCMC analysis for this paper

```
##Conduct the TimeOpt and TimtOptMCMC analysis to obtain the precessional constant index (k)
### GR data from Li et al (2018 EPSL), GR series 10-72 m (245 Ma)
library(astrochron)
data=read();
data1=iso(data,xmin=10,xmax=72);
data1=trim(data1,c=2);
data1=noKernel(data1,smooth=0.1);
### Interpolate the data to the median sampling interval
data1=linterp(data1)
###Determine nominal precession and eccentricity periods,then conduct nominal timeOpt analysis
targetTot=calcPeriods(g=c(5.525000,7.455000,17.300000,17.850000,4.257455),k=54.5,output=2);
targetE=sort(targetTot[1:5],decreasing=T);
targetP=sort(targetTot[6:10],decreasing=T);
###run nominal timeOpt and output sedimentation rate grid and fit
res1=timeOpt(data1,sedmin=4,sedmax=7,numsed=100,targetE=targetE,targetP=targetP,flow=1/23
,fhigh=1/17,roll=10^7,limit=T,output=1);
###output optimal time series, bandpassed series, amplitude envelope and TimeOpt-reconstructed eccentricity
res2=timeOpt(data1,sedmin=4,sedmax=7,numsed=100,targetE=targetE,targetP=targetP,flow=1/23
,fhigh=1/17,roll=10^7,limit=T,output=2);
###perform nominal timeOpt significance testing
simres=timeOptSim(data1,sedmin=4,sedmax=7,numsed=100,targetE=targetE,targetP=targetP,flow
=1/23,fhigh=1/17,roll=10^7,numsim=1000,output=2,ncores=4);
###plot summary figure
timeOptPlot(data1,res1,res2,simres,flow=1/23,fhigh=1/17,fitR=0.20783,roll=10^7,targetE=targetE
,targetP=targetP,xlab="Height(cm)",ylab="GR",verbose=T);
###run a single timeOptMCMC chain (100 chains)
res=timeOptMCMC(data1,sedmin=4,sedmax=7,sedstart=5.94,gAve=c(5.525000,7.455000,17.300
000,17.850000,4.257455),  gSd=c(0.12500,0.01500,0.150005,0.15000,0.00002),gstart=c(-1,-1,-1,-
1,-1),kAve=54.5,kSd=2.5,kstart=-
1,rhomin=0,rhomax=0.9999,rhostart=1,sigmamin=NULL,sigmamax=NULL,sigmastart=-
1,nsamples=200000,
iopt=1,epsilon=c(0.2,0.2,0.35,0.35,0.8,0.85,0.6,0.35,0.9,0.35,0.9)/40,ran=T,burnin=-1,savefile = F);
### output the TimeOptMCMC results
write.table(res,file="Li_GR_TimeOptMCMC_results.csv",sep=",",row.names=FALSE)


###TimeOptMCMC analysis the Ji251 NGR series from Huang et al., 2020_P3 (290Ma)
library(astrochron);
```

```
###Obtain the target dataset
ji=read()
ji251=iso(ji,xmin=3650,xmax=3770);
ji1=trim(ji251,c=3);
ji2=linterp(ji1,dt=0.5);
###Determine nominal precession and eccentricity periods,then conduct nominal timeOpt analysis
targetTot=calcPeriods(g=c(5.525000,7.455000,17.300000,17.850000,4.257455),k=55,output=2);
targetE=sort(targetTot[1:5],decreasing=T);
targetP=sort(targetTot[6:10],decreasing=T);
###run nominal timeOpt and output sedimentation rate grid and fit
res1=timeOpt(ji2,sedmin=2,sedmax=18,numsed=100,targetE=targetE,targetP=targetP,flow=1/23,f
high=1/16,roll=10^7,limit=T,output=1);
###output optimal time series, bandpassed series, amplitude envelope and TimeOpt-reconstructed
eccentricity
res2=timeOpt(ji2,sedmin=2,sedmax=18,numsed=100,targetE=targetE,targetP=targetP,flow=1/23,f
high=1/16,roll=10^7,limit=T,output=2);
###perform nominal timeOpt significance testing
simres=timeOptSim(ji2,sedmin=2,sedmax=18,numsed=100,targetE=targetE,targetP=targetP,flow=
1/23,fhigh=1/16,roll=10^7,numsim=2000,output=2,ncores=6);
###plot summary figure
timeOptPlot(ji2,res1,res2,simres,flow=1/23,fhigh=1/16,fitR=0.19915,roll=10^7,targetE=targetE,ta
rgetP=targetP,xlab="Height(m)",ylab="NGR",verbose=T);
###run a single timeOptMCMC chain (150 chain)
res=timeOptMCMC(ji2,sedmin=2,sedmax=18,sedstart=9.78,gAve=c(5.525000,7.455000,17.3000
00,17.850000,4.257455),gSd=c(0.12500,0.01500,0.150005,0.15000,0.00002),gstart=c(-1,-1,-1,-1,-
1),kAve=55,kSd=3,kstart=-1,rhomin=0,rhomax=0.9999,rhostart=-
1,sigmamin=NULL,sigmamax=NULL,sigmastart=1,nsamples=100000,iopt=1,epsilon=c(0.2,0.2,0
.35,0.35,0.8,0.85,0.6,0.35,0.9,0.35,0.9)/20,ran=T,burnin=-1);
### output the TimeOptMCMC results
write.table(res,file="Huang_NGR_TimeOptMCMC_results.csv",sep=",",row.names=FALSE)


### Data from De Vleeschouwer et al (2017 Nature Communications) H32_MS series, 176-900cm
(~375 Ma)
###(1)load the Astrochron package
library(astrochron);
###(2) Obtain the target dataset
data=read();
data1=iso(data,xmin=176,xmax=900);
# Convert depth from cm to m
data1[1]=data1[1]/100
data1=noKernel(data1,smooth=0.1);
data1=trim(data1,c=1.5);
###(3) Interpolate the data to the median sampling interval
```

```
data1=linterp(data1);
###Determine nominal precession and eccentricity periods,then conduct nominal timeOpt analysis
targetTot=calcPeriods(g=c(5.525000,7.455000,17.300000,17.850000,4.257455),k=58,output=2);
targetE=sort(targetTot[1:5],decreasing=T);
targetP=sort(targetTot[6:10],decreasing=T);
###run nominal timeOpt and output sedimentation rate grid and fit
res1=timeOpt(data1,sedmin=0.7,sedmax=1,numsed=100,targetE=targetE,targetP=targetP,flow=1/
23,fhigh=1/16,roll=10^7,limit=T,output=1);
###output optimal time series, bandpassed series, amplitude envelope and TimeOpt-reconstructed
eccentricity
res2=timeOpt(data1,sedmin=0.7,sedmax=1,numsed=100,targetE=targetE,targetP=targetP,flow=1/
23,fhigh=1/16,roll=10^7,limit=T,output=2);
###perform nominal timeOpt significance testing
simres=timeOptSim(data1,sedmin=0.7,sedmax=1,numsed=100,targetE=targetE,targetP=targetP,fl
ow=1/23,fhigh=1/16,roll=10^7,numsim=2000,output=2,ncores=6);
###plot summary figure
timeOptPlot(data1,res1,res2,simres,flow=1/23,fhigh=1/16,fitR=0.18966,roll=10^7,targetE=targetE
,targetP=targetP,xlab="Height(m)",ylab="MS",verbose=T);
###run a single timeOptMCMC chain (200 chain)
res=timeOptMCMC(data1,sedmin=0.7,sedmax=1,sedstart=0.83,gAve=c(5.525000,7.455000,17.30
0000,17.850000,4.257455),gSd=c(0.12500,0.01500,0.150005,0.15000,0.00002),gstart=c(-1,-1,-1,-
1,-1),kAve=58,kSd=4,kstart=-1,rhomin=0,rhomax=0.9999,rhostart=-
1,sigmamin=NULL,sigmamax=NULL,sigmastart=-1,nsamples=100000,
iopt=1,epsilon=c(0.2,0.2,0.35,0.35,0.8,0.85,0.6,0.35,0.9,0.35,0.9)/20,ran=T,burnin=-1);
### output the TimeOptMCMC results
write.table(res,file="David_MS_375Ma_TimeOptMCMC_results.csv",sep=",",row.names=FALS
E)

### Data from Da Silva et al (2016 EPSL) Požár-CS section_MS series (106.7-114m), (~410 Ma).
###(1)load the Astrochron package
library(astrochron);
###(2) Obtain the target dataset
data=read();
data1=iso(data,xmin=106.7,xmax=114);
data1=noKernel(data1,smooth=0.5);
data1=trim(data1,c=2);
###(3) Interpolate the data to the median sampling interval
data1=linterp(data1);
###Determine nominal precession and eccentricity periods,then conduct nominal timeOpt analysis
targetTot=calcPeriods(g=c(5.525000,7.455000,17.300000,17.850000,4.257455),k=58,output=2);
targetE=sort(targetTot[1:5],decreasing=T);
targetP=sort(targetTot[6:10],decreasing=T);
###run nominal timeOpt and output sedimentation rate grid and fit
res1=timeOpt(data1,sedmin=0.2,sedmax=1,numsed=100,targetE=targetE,targetP=targetP,flow=1/
```

```
25,fhigh=1/16,roll=10^7,limit=T,output=1);
###output optimal time series, bandpassed series, amplitude envelope and TimeOpt-reconstructed
eccentricity
res2=timeOpt(data1,sedmin=0.2,sedmax=1,numsed=100,targetE=targetE,targetP=targetP,flow=1/
25,fhigh=1/16,roll=10^7,limit=T,output=2);
###perform nominal timeOpt significance testing
simres=timeOptSim(data1,sedmin=0.2,sedmax=1,numsed=100,targetE=targetE,targetP=targetP,fl
ow=1/25,fhigh=1/16,roll=10^7,numsim=2000,output=2,ncores=6);
###plot summary figure
timeOptPlot(data1,res1,res2,simres,flow=1/25,fhigh=1/16,fitR=0.162,roll=10^7,targetE=targetE,ta
rgetP=targetP,xlab="Height(m)",ylab="MS",verbose=T);
###run a single timeOptMCMC chain (150 chain)
res=timeOptMCMC(data1,sedmin=0.2,sedmax=1,sedstart=0.83,gAve=c(5.525000,7.455000,17.30
0000,17.850000,4.257455),gSd=c(0.12500,0.01500,0.150005,0.15000,0.00002),gstart=c(-1,-1,-1,-
1,-1),kAve=58,kSd=4,kstart=-1,rhomin=0,rhomax=0.9999,rhostart=-
1,sigmamin=NULL,sigmamax=NULL,sigmastart=-1,nsamples=200000,
iopt=1,epsilon=c(0.2,0.2,0.35,0.35,0.8,0.85,0.6,0.35,0.9,0.35,0.9)/20,ran=T,burnin=-1);
### output the TimeOptMCMC results
write.table(res,file="Dasilva_MS_410Ma_TimeOptMCMC_results.csv",sep=",",row.names=FAL
SE)

### Data from Sinnesael et al (2021 Geology) 550-900 m K% time series (~448 Ma)
###(2) Obtain the target dataset
library(astrochron);
data=read()
data1=noKernel(data,smooth=0.1);
data1=iso(data1,xmin=550,xmax=900);
data1=trim(data1,c=1.5);
data2=linterp(data1,dt=2);
###Determine nominal precession and eccentricity periods,then conduct nominal timeOpt analysis
targetTot=calcPeriods(g=c(5.525000,7.455000,17.300000,17.850000,4.257455),k=59,output=2);
targetE=sort(targetTot[1:5],decreasing=T);
targetP=sort(targetTot[6:10],decreasing=T);
###run nominal timeOpt and output sedimentation rate grid and fit
res1=timeOpt(data2,sedmin=10,sedmax=60,numsed=100,targetE=targetE,targetP=targetP,flow=1/
23,fhigh=1/15,roll=10^7,limit=T,output=1);
###output optimal time series, bandpassed series, amplitude envelope and TimeOpt-reconstructed
eccentricity
res2=timeOpt(data2,sedmin=10,sedmax=60,numsed=100,targetE=targetE,targetP=targetP,flow=1/
23,fhigh=1/15,roll=10^7,limit=T,output=2);
###perform nominal timeOpt significance testing
simres=timeOptSim(data2,sedmin=10,sedmax=60,numsed=100,targetE=targetE,targetP=targetP,fl
ow=1/23,fhigh=1/15,roll=10^7,numsim=2000,output=2,ncores=6);
###plot summary figure
```

```
timeOptPlot(data2,res1,res2,simres,flow=1/23,fhigh=1/15,fitR=0.21654,roll=10^7,targetE=targetE
,targetP=targetP,xlab="Height(m)",ylab="K",verbose=T);
###run a single timeOptMCMC chain (100 chain)
res=timeOptMCMC(data2,sedmin=10,sedmax=60,sedstart=47.3,gAve=c(5.525000,7.455000,17.3
00000,17.850000,4.257455),gSd=c(0.12500,0.01500,0.150005,0.15000,0.00002),gstart=c(-1,-1,-
1,-1,-1),kAve=59,kSd=4,kstart=-1,rhomin=0,rhomax=0.9999,rhostart=-
1,sigmamin=NULL,sigmamax=NULL,sigmastart=1,nsamples=200000,
iopt=1,epsilon=c(0.2,0.2,0.35,0.35,0.8,0.85,0.6,0.35,0.9,0.35,0.9)/20,ran=T,burnin=-1);
### output the TimeOptMCMC results
write.table(res,file="Sinnesael_K_445Ma_TimeOptMCMC_results.csv",sep=",",row.names=FAL
SE)

### Data from Ma et al (2019 P3) LJS Ca% time series (~470 Ma)

###(2) Obtain the target dataset
library(astrochron);
data=read()
data1=iso(data,xmin=45,xmax=62)
data1=noKernel(data1,smooth=0.5);
data1=trim(data1,c=1.5);
data2=linterp(data1,dt=0.1);
###Determine nominal precession and eccentricity periods,then conduct nominal timeOpt analysis
targetTot=calcPeriods(g=c(5.525000,7.455000,17.300000,17.850000,4.257455),k=59,output=2);
targetE=sort(targetTot[1:5],decreasing=T);
targetP=sort(targetTot[6:10],decreasing=T);
###run nominal timeOpt and output sedimentation rate grid and fit
res1=timeOpt(data2,sedmin=0.1,sedmax=1.8,numsed=100,targetE=targetE,targetP=targetP,flow=1
/22,fhigh=1/15,roll=10^7,limit=T,output=1);
###output optimal time series, bandpassed series, amplitude envelope and TimeOpt-reconstructed
eccentricity
res2=timeOpt(data2,sedmin=0.1,sedmax=1.8,numsed=100,targetE=targetE,targetP=targetP,flow=1
/22,fhigh=1/15,roll=10^7,limit=T,output=2);
###perform nominal timeOpt significance testing
simres=timeOptSim(data2,sedmin=0.1,sedmax=1.8,numsed=100,targetE=targetE,targetP=targetP,f
low=1/22,fhigh=1/15,roll=10^7,numsim=2000,output=2,ncores=6);
###plot summary figure
timeOptPlot(data2,res1,res2,simres,flow=1/22,fhigh=1/15,fitR=0.12135,roll=10^7,targetE=targetE
,targetP=targetP,xlab="Height(m)",ylab="Ca",verbose=T);
###run a single timeOptMCMC chain (50 chain)
res=timeOptMCMC(data2,sedmin=0.1,sedmax=1.8,sedstart=1.59,gAve=c(5.525000,7.455000,17.
300000,17.850000,4.257455),gSd=c(0.12500,0.01500,0.150005,0.15000,0.00002),gstart=c(-1,-1,-
1,-1,-1),kAve=59,kSd=5,kstart=-1,rhomin=0,rhomax=0.9999,rhostart=-
1,sigmamin=NULL,sigmamax=NULL,sigmastart=1,nsamples=600000,
iopt=1,epsilon=c(0.2,0.2,0.35,0.35,0.8,0.85,0.6,0.35,0.9,0.35,0.9)/40,ran=T,burnin=-1);
```

```
### output the TimeOptMCMC results
write.table(res,file="Ma_Ca_470Ma_TimeOptMCMC_results.csv",sep=",",row.names=FALSE)

###### Data from Sorensen et al (2020 EPSL) S% (83-85.5m) time series (~493 Ma)
library(astrochron);
###Obtain the target dataset
Soren=read();
###Interpolate the data to the median sampling interval
Soren1=linterp(Soren,dt=0.01);
Soren2=iso(Soren1,xmin=83, xmax=85.5);
Soren2=trim(Soren2,c=1.5);
Soren2=linterp(Soren2,dt=0.02);
###Determine nominal precession and eccentricity periods,then conduct nominal timeOpt analysis
targetTot=calcPeriods(g=c(5.525000,7.455000,17.300000,17.850000,4.257455),k=59,output=2);
targetE=sort(targetTot[1:5],decreasing=T);
targetP=sort(targetTot[6:10],decreasing=T);
###run nominal timeOpt and output sedimentation rate grid and fit
res1=timeOpt(Soren2,sedmin=0.1,sedmax=0.4,numsed=100,targetE=targetE,targetP=targetP,flow
=1/22,fhigh=1/15,roll=10^7,limit=T,output=1);
###output optimal time series, bandpassed series, amplitude envelope and TimeOpt-reconstructed
eccentricity
res2=timeOpt(Soren2,sedmin=0.1,sedmax=0.4,numsed=100,targetE=targetE,targetP=targetP,flow
=1/22,fhigh=1/15,roll=10^7,limit=T,output=2);
###perform nominal timeOpt significance testing
simres=timeOptSim(Soren2,sedmin=0.1,sedmax=0.4,numsed=100,targetE=targetE,targetP=target
P,flow=1/22,fhigh=1/15,roll=10^7,numsim=2000,output=2,ncores=6);
###plot summary figure
timeOptPlot(Soren2,res1,res2,simres,flow=1/22,fhigh=1/15,fitR=0.18408,roll=10^7,targetE=targe
tE,targetP=targetP,xlab="Height(m)",ylab="S",verbose=T);
###run a single timeOptMCMC chain (100 chain)
res=timeOptMCMC(Soren2,sedmin=0.1,sedmax=0.5,sedstart=0.34,gAve=c(5.525000,7.455000,1
7.300000,17.850000,4.257455),gSd=c(0.12500,0.01500,0.150005,0.15000,0.00002),gstart=c(-1,-
1,-1,-1,-1),kAve=59,kSd=5,kstart=-1,rhomin=0,rhomax=0.9999,rhostart=-
1,sigmamin=NULL,sigmamax=NULL,sigmastart=1,nsamples=200000,
iopt=1,epsilon=c(0.2,0.2,0.35,0.35,0.8,0.85,0.6,0.35,0.9,0.35,0.9)/40,ran=T,burnin=-1);
### output the TimeOptMCMC results
write.table(res,file="Sorensen_S%_493Ma_TimeOptMCMC_results.csv",sep=",",row.names=FA
LSE)

### Data from Li et al (2022, Global and Planetary Changes) MS time series (570 Ma)
library(astrochron);
###Obtain the target dataset
Li=read();
### Interpolate the data to the median sampling interval
```

```
Li=linterp(Li);
Li_1=iso(Li,xmin=26,xmax=33);
Li_2=noKernel(Li_1,smooth=0.5);
Li_3=trim(Li_2,c=1.5);
Li_4=linterp(Li_3,dt=0.03);
###Determine nominal precession and eccentricity periods,then conduct nominal timeOpt analysis
targetTot=calcPeriods(g=c(5.525000,7.455000,17.300000,17.850000,4.257455),k=60,output=2);
targetE=sort(targetTot[1:5],decreasing=T);
targetP=sort(targetTot[6:10],decreasing=T);
###run nominal timeOpt and output sedimentation rate grid and fit
res1=timeOpt(Li_4,sedmin=0.5,sedmax=0.9,numsed=100,targetE=targetE,targetP=targetP,flow=1/
21,fhigh=1/15,roll=10^7,limit=T,output=1);
###output optimal time series, bandpassed series, amplitude envelope and TimeOpt-reconstructed
eccentricity
res2=timeOpt(Li_4,sedmin=0.5,sedmax=0.9,numsed=100,targetE=targetE,targetP=targetP,flow=1/
21,fhigh=1/15,roll=10^7,limit=T,output=2);
###perform nominal timeOpt significance testing
simres=timeOptSim(Li_4,sedmin=0.5,sedmax=0.9,numsed=100,targetE=targetE,targetP=targetP,fl
ow=1/21,fhigh=1/15,roll=10^7,numsim=2000,output=2,ncores=6);
###plot summary figure
timeOptPlot(Li_4,res1,res2,simres,flow=1/21,fhigh=1/15,fitR=0.1889,roll=10^7,targetE=targetE,t
argetP=targetP,xlab="Height(m)",ylab="MS",verbose=T);
###run a single timeOptMCMC chain (100 chain)
res=timeOptMCMC(Li_4,sedmin=0.5,sedmax=0.9,sedstart=0.77,gAve=c(5.525000,7.455000,17.3
00000,17.850000,4.257455),gSd=c(0.12500,0.01500,0.150005,0.15000,0.00002),gstart=c(-1,-1,-
1,-1,-1),kAve=60,kSd=5,kstart=-1,rhomin=0,rhomax=0.9999,rhostart=-
1,sigmamin=NULL,sigmamax=NULL,sigmastart=1,nsamples=200000,
iopt=1,epsilon=c(0.2,0.2,0.35,0.35,0.8,0.85,0.6,0.35,0.9,0.35,0.9)/20,ran=T,burnin=-1);
### output the TimeOptMCMC results
write.table(res,file="Li_MS_570Ma_TimeOptMCMC_results.csv",sep=",",row.names=FALSE)
```

References

1. J. Laskar et al., A long-term numerical solution for the insolation quantities of the Earth. Astron. Astrophys. 428, 261–285 (2004).
2. D. De Vleeschouwer et al., North Atlantic Drift Sediments Constrain Eocene Tidal Dissipation and the Evolution of the Earth-Moon System. Paleoceanography and Paleoclimatology 38 (2023).
3. S. R. Meyers, A. Malinverno, Proterozoic Milankovitch cycles and the history of the solar system. Proc. Natl. Acad. Sci. U.S.A. 115, 6363–6368 (2018).
4. N. J. de Winter et al., Subdaily-Scale Chemical Variability in a Torreites Sanchezi Rudist Shell: Implications for Rudist Paleobiology and the Cretaceous Day-Night Cycle. Paleoceanography and Paleoclimatology 35 (2020).
5. M. Li et al., Astrochronology of the Anisian stage (Middle Triassic) at the Guandao reference section, South China. Earth Planet. Sci. Lett. 482, 591–606 (2018).
6. M. Zhou et al., Empirical Reconstruction of Earth-Moon and Solar System Dynamical Parameters for the Past 2.5 Billion Years From Cyclostratigraphy. Geophysical Research Letters 49 (2022).
7. H. Huang et al., Astronomical forcing of Middle Permian terrestrial climate recorded in a large paleolake in northwestern China. Palaeogeography, Palaeoclimatology, Palaeoecology 550, 109735 (2020).
8. D. De Vleeschouwer et al., Timing and pacing of the Late Devonian mass extinction event regulated by eccentricity and obliquity. Nature Communications 8 (2017).
9. C. Zeeden, J. Laskar, D. V Vleeschouwer, D. Pas, A.C. Da Silva, Earth's rotation and Earth-Moon distance in the Devonian derived from multiple geological records. Earth Planet. Sci. Lett. 621, 118348 (2023).
10. A. C. Da Silva et al., Refining the Early Devonian time scale using Milankovitch cyclicity in Lochkovian–Pragian sediments (Prague Synform, Czech Republic). Earth and Planetary Science Letters 455, 125–139 (2016).
11. M. Sinnesael et al., Precession-driven climate cycles and time scale prior to the Hirnantian glacial maximum. Geology (2021).
12. K. Ma, R. Li, L.A. Hinnov, Y. Gong, Conodont biostratigraphy and astronomical tuning of the Lower-Middle Ordovician Liangjiashan (North China) and Huanghuachang (South China) marine sections. Palaeogeography Palaeoclimatology Palaeoecology 528, 272–287 (2019).
13. A. L. Sørensen et al., Astronomically forced climate change in the late Cambrian. Earth Planet. Sci. Lett. 548, 116475 (2020).
14. J. Fang., H. Wu., Q. Fang., M. Shi., S. Zhang., T. Yang., H. Li., L. Cao., Cyclostratigraphy of the global stratotype section and point (GSSP) of the basal Guzhangian Stage of the Cambrian Period. Palaeogeography, Palaeoclimatology, Palaeoecology, 540 (2019).
15. T. Zhang et al., Orbitally-paced climate change in the early Cambrian and its implications for the history of the Solar System. Earth Planet. Sci. Lett. 583, 117420 (2022).

16. H. Li et al., Astrochronologic calibration of the Shuram carbon isotope excursion with new data from South China. Global and Planetary Change 209, 103749 (2022).
17. C. P. Sonett, M. A. Chan, Neoproterozoic Earth-Moon dynamics: Rework of the 900 Ma Big Cottonwood Canyon tidal laminae. Geophysical Research Letters 25, 539–542, (1998).
18. M. L. Lantink, J. Davies, M. Ovtcharova, F. J. Hilgen, Milankovitch cycles in banded iron formations constrain the Earth-Moon system 2.46 billion years ago. Proc Natl Acad Sci U.S.A. 119, e2117146119 (2022).
19. D. Waltham, Milankovitch period uncertainties and their impact on cyclostratigraphy. J. Sediment. Res. 85, 990–998 (2015).
20. M. Farhat, P. Auclair-Desrotour, G. Boue, J. Laskar, The resonant tidal evolution of the Earth-Moon distance. Astron. Astrophys. 665, L1 (2022).
21. C. R. Scotese, An Atlas of Phanerozoic Paleogeographic Maps: The Seas Come In and the Seas Go Out. Annual Review of Earth and Planetary Sciences 49, 679–728 (2021).
22. W. Cao, C. T. A. Lee, J.S. Lackey, Episodic nature of continental arc activity since 750 Ma: a global compilation. Earth Planet. Sci. Lett. 461, 85–95 (2017).
23. S. E. Peter and J.M. Husson, Sediment cycling on continental and oceanic crust. Geology (2016).
24. R. D. Nance, J.B. Murphy, M. Santosh, The supercontinent cycle: A retrospective essay. Gondwana Research 25, 4–29 (2014).
25. B.J.W. Mills, C.R. Scotese, N.G. Walding, G.A. Shields, T.M. Lenton, Elevated CO2 degassing rates prevented the return of Snowball Earth during the Phanerozoic. Nat Commun. 8, 1110 (2017).
26. B. J. Mills, A. J. Krause, I. Jarvis, B. D. Cramer, Evolution of atmospheric O2 through the Phanerozoic, Revisited. Annual Review of Earth and Planetary Sciences 51, 253–76 (2023).
27. T. W. Lyons, C.T. Reinhard, N.J. Planavsky, The rise of oxygen in Earth's early ocean and atmosphere. Nature 506, 307–315 (2014).
28. J. Fan et al., A high-resolution summary of Cambrian to Early Triassic marine invertebrate biodiversity. Science 367, 272–277 (2020).
